# African based researchers’ output on models for the transmission dynamics of infectious diseases and public health interventions: A scoping review

**DOI:** 10.1371/journal.pone.0250086

**Published:** 2021-05-06

**Authors:** Olatunji O. Adetokunboh, Zinhle E. Mthombothi, Emanuel M. Dominic, Sylvie Djomba-Njankou, Juliet R. C. Pulliam

**Affiliations:** 1 DSI-NRF Centre of Excellence in Epidemiological Modelling and Analysis (SACEMA), Stellenbosch University, Stellenbosch, South Africa; 2 Division of Epidemiology and Biostatistics, Department of Global Health, Stellenbosch University, Cape Town, South Africa; Texas A&M University College Station, UNITED STATES

## Abstract

**Background:**

Applied epidemiological models are used in predicting future trends of diseases, for the basic understanding of disease and health dynamics, and to improve the measurement of health indicators. Mapping the research outputs of epidemiological modelling studies concerned with transmission dynamics of infectious diseases and public health interventions in Africa will help to identify the areas with substantial levels of research activities, areas with gaps, and research output trends.

**Methods:**

A scoping review of applied epidemiological models of infectious disease studies that involved first or last authors affiliated to African institutions was conducted. Eligible studies were those concerned with the transmission dynamics of infectious diseases and public health interventions. The review was consistent with the Preferred Reporting Items for Systematic Reviews and Meta-Analysis (PRISMA) extension for scoping reviews. Four electronic databases were searched for peer-reviewed publications up to the end of April 2020.

**Results:**

Of the 5927 publications identified, 181 met the inclusion criteria. The review identified 143 publications with first authors having an African institutional affiliation (AIA), while 81 had both first and last authors with an AIA. The publication authors were found to be predominantly affiliated with institutions based in South Africa and Kenya. Furthermore, human immunodeficiency virus, malaria, tuberculosis, and Ebola virus disease were found to be the most researched infectious diseases. There has been a gradual increase in research productivity across Africa especially in the last ten years, with several collaborative efforts spread both within and beyond Africa.

**Conclusions:**

Research productivity in applied epidemiological modelling studies of infectious diseases may have increased, but there remains an under-representation of African researchers as leading authors. The study findings indicate a need for the development of research capacity through supporting existing institutions in Africa and promoting research funding that will address local health priorities.

## Introduction

One of the visions of the 2030 Agenda for Sustainable Development Goals is access to healthcare with the assurance of physical, mental, and social well-being [1]. Accomplishing this primarily requires achieving universal health coverage, access to effective and safe medicines, and vaccines for all age groups [1]. Furthermore, through the Agenda 2063 framework, African leaders aspire to achieve a high standard of living, good quality of life, sound health and well-being for Africans by 2063 [2]. Supportive research and development of interventions aimed at strengthening health systems and generating predictions of implementation outcomes are essential for the attainment of these goals and agendas. However, despite the progress made in achieving healthy lives and well-being for all, the world still records about six million under-five deaths per year. Communicable and non-communicable diseases remain the leading causes of morbidity and mortality in Africa, with human immunodeficiency virus (HIV) infection, tuberculosis (TB), malaria, cardiovascular conditions, and cancers accounting for a considerable proportion of all illnesses and deaths each year [3, 4].

Achieving healthy lives and well-being for all requires the full commitment of all stakeholders such as the governments, civil societies, the private sector, academia, and research institutions [4]. Vibrant groups of researchers are engaged in ongoing activities targeted at developing solutions, development of novel medical products, and strengthening of the health systems [5]. African countries are primarily working towards being amongst the best performers in global quality of life measures via strategies such as investment in research and innovation [2]. For instance, the South African National Strategic Plan for HIV, TB, and sexually transmitted infections (STIs) 2017–2022 aims to strengthen strategic research activities that will create validated evidence [6]. Many African countries, including South Africa, Nigeria, and Egypt, are investing in research and development [6, 7].

Epidemiological modelling is a research methodology used by various public health and mathematical researchers [8, 9]. Epidemiological models are used in predicting future trends of diseases, for the basic understanding of disease and health dynamics, and to improve the measurement and interpretation of health indicators at both individual and population levels [8, 9]. These models also help in developing study designs and intervention roll-out strategies, evaluation of the impact of public health interventions, and assessment of future public health risks. Epidemiological modelling can inform decisions via the analysis and assessment of various policy options for policymaking. Mechanistic epidemiological models have increasingly gained prominence as tools for understanding the transmission dynamics of diseases and the evaluation of the possible impacts of control programmes targeted at decreasing morbidity and mortality [10].

Studies on epidemiological and global health research outputs in Africa show an increasing trend concerning research volume from 172 to 1086 peer-reviewed articles per annum between 1991 and 2010; however, most of the outputs are limited to a few countries, such as South Africa, Kenya and Nigeria, thereby showcasing vast research inequality among African countries [11, 12]. As a continent with a high burden of disease, Africa requires expertise in epidemiological modelling. African researchers are needed to work on diseases of public health importance. Local experts need to lead the investigations of public health issues within their environment due to their knowledge and understanding of local social and contextual issues. This will also encourage stakeholders’ buy-in and sustainability of research projects, as well as supporting an increase in the pool of trained African public health researchers. The involvement of health researchers is critical for innovation and the development of methods to improve the quality of healthcare in Africa [13].

Mapping the research outputs of applied epidemiological modelling of infectious disease studies that are directly linked to specific public health interventions will aid the identification of geographical regions within Africa with substantial levels of research activities, as well as areas with research gaps. Furthermore, these mappings will highlight general research output trends and bring to light disease modelling themes that are already well explored. To this end, a scoping review of applied epidemiological models of infectious diseases that can be linked to specific public health interventions and involved first or last African institutional affiliated (AIA) authors was conducted. This review will focus on mechanistic models which could be defined as “*hypothesized relationship between the variables in a dataset where the nature of the relationship is specified in terms of the biological processes that are thought to have given rise to the data*. *The parameters in the mechanistic model all have biological definitions and so they can be measured independently of the dataset referenced”* [14].

This review’s findings are expected to guide the development of research, funding, and capacity development priorities for the African continent. It will identify the level of collaboration between African and non-African institutions with respect to applied epidemiological modelling of infectious diseases.

## Methods and materials

A protocol was developed following the Preferred Reporting Items for Systematic Reviews and Meta-Analysis extension for scoping reviews (PRISMA-ScR) guidelines [15]. This study was registered with the Open Science Framework (https://osf.io/2rs4j/).

### I. Identification of the research objectives

#### Primary objective

To summarise existing peer-reviewed publications of applied epidemiological modelling studies on infectious diseases in Africa.

### Secondary objectives

- To identify the infectious diseases of interest.
- To identify the first and last authors affiliated with African institutions and/or research centres. The African affiliated researchers are those who contributed to research work in the institutions based in Africa and may not necessarily reside in Africa.
- To determine the collaborative efforts among researchers within Africa and beyond.
- To investigate the publication trends of epidemiological modelling studies on infectious diseases in Africa.
- To assess the expansion (or lack thereof) of applied epidemiological modelling on infectious diseases within Africa.
- To identify geographic hotspots of applied epidemiological modelling on infectious diseases within Africa.

### II. Identification of relevant studies for inclusion

We were interested in published, peer-reviewed journal articles and restricted the literature search to English Language. We used the following electronic databases: PubMed, Scopus, Web of Science and African Journals Online. The four electronic databases were searched for peer-reviewed publications for all African countries up to the end of April 2020. The researchers also did a hand search of references from the list of included studies. The database outputs were uploaded to the Mendeley reference management tool (https://www.mendeley.com) to remove duplicated records.

#### Eligibility criteria

Inclusion criteria

- Primary studies with the first or last authors having an African institutional affiliation (AIA). This review is interested in knowing the African affiliated researchers involved in research leadership and management, which could be inferred by the first or the last authorship positioning.
- Studies published in peer-reviewed journals.
- Studies whose primary focus was public health interventions in humans.

Exclusion criteria

1. Review articles.
2. Studies that do not focus on applied epidemiological modelling of infectious diseases.
3. Cost-effectiveness models that do not have an underlying mechanistic model.
4. Statistical analyses that do not have an underlying mechanistic model.
5. Theoretical modelling exercises that do not link to public health applications.
6. Studies that do not include authors with a first or last African institutional affiliation.
7. Articles published in languages other than English.

### III. Study selection

Four researchers (OAA, ZEM, ED and SDN) independently screened the titles and abstracts from the exported database outputs and saved these in the Rayyan QCRI, the Systematic Reviews web app [16]. Studies that did not meet the inclusion criteria were excluded. From the eligible studies, the full articles were independently screened by OAA, ZEM, ED and SDN. Discrepancies between the reviewers were resolved through consensus.

The following search terms were employed for the search: ("transmission dynamics" OR "mathematical model" OR "transmission model" OR "dynamic model" OR "simulation model" OR "stochastic model" OR "deterministic model" OR "mechanistic model" OR "computer model") AND ("infectious disease" OR disease OR "communicable disease") AND (Algeria OR Angola OR Benin OR Botswana OR "Burkina Faso" OR Burundi OR "Cabo Verde" OR "Cape Verde" OR Cameroon OR "Central African Republic" OR Chad OR Comoros OR "Congo, Democratic Republic of the" OR "Democratic Republic of Congo" OR "Congo, Republic of the" OR "Republic of Congo" OR Congo OR "Cote d’Ivoire" OR "Ivory Coast" OR Djibouti OR Egypt OR "Equatorial Guinea" OR Eritrea OR Eswatini OR Swaziland OR Ethiopia OR Gabon OR Gambia OR Ghana OR Guinea OR Guinea-Bissau OR Kenya OR Lesotho OR Liberia OR Libya OR Madagascar OR Malawi OR Mali OR Mauritania OR Mauritius OR Morocco OR Mozambique OR Namibia OR Niger OR Nigeria OR Rwanda OR "Sao Tome and Principe" OR Senegal OR Seychelles OR "Sierra Leone" OR Somalia OR "South Africa" OR "South Sudan" OR Sudan OR Tanzania OR Togo OR Tunisia OR Uganda OR Zambia OR Zimbabwe OR "Sub-Saharan Africa" OR Africa)

**Fig 1:**
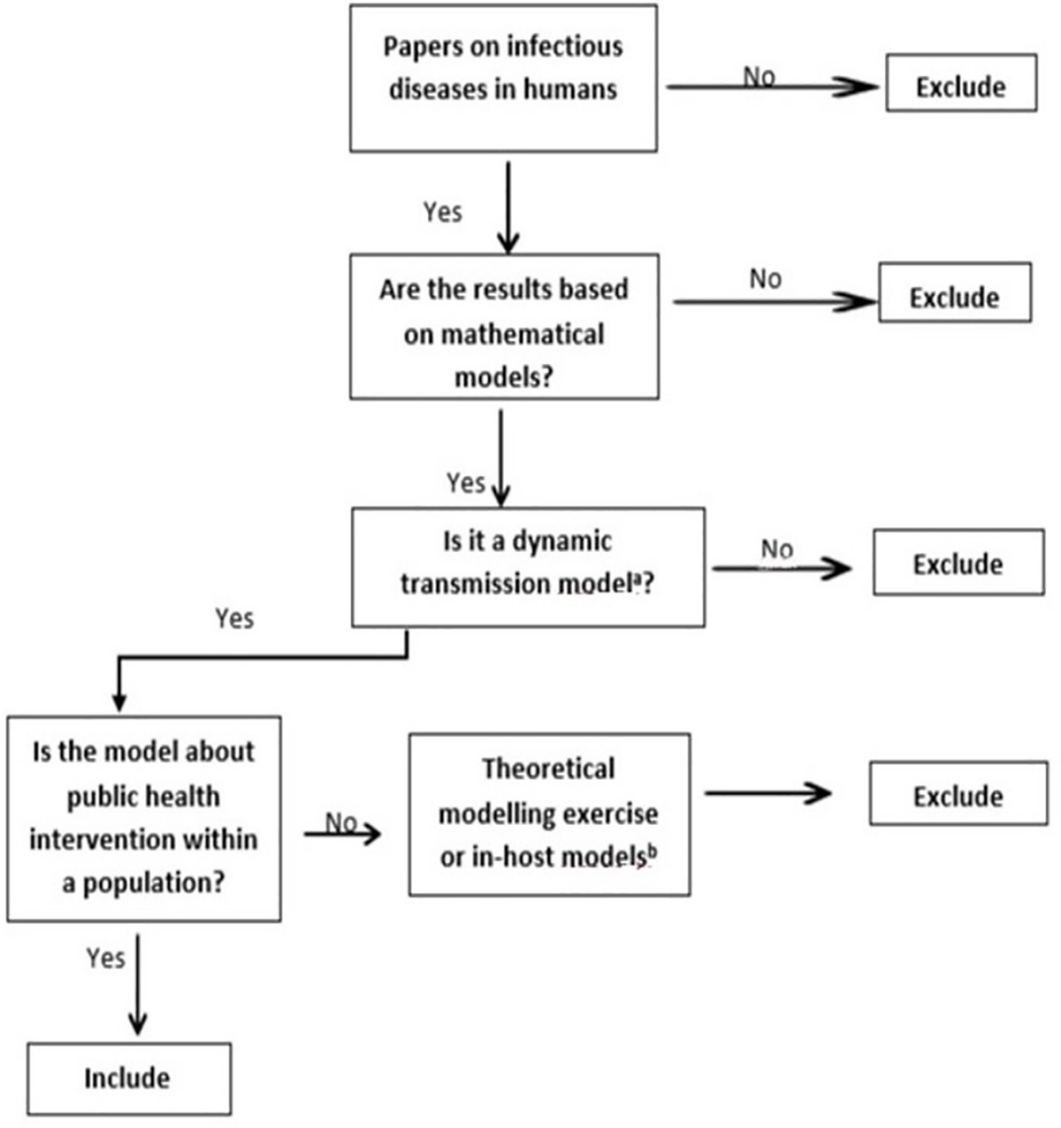
The screening algorithm used to determine the eligibility of literature for review. ^a^ Dynamic transmission models are mathematical models in which transmission is determined endogenously [17]. ^b^ Theoretical models are used to understand transmission dynamics but informing to public health action or policy is not their primary aim [17].

The algorithm used to summarise the screening process follows hereafter in Fig 1.

### IV. Data extraction

The required information was extracted from the included studies using a Microsoft Excel pre-defined data extraction form. The data extraction form captured the following variables: first author’s name, last author’s name, year of publication, African affiliations, country of affiliation, journal, study title, diseases of interest and international collaborative efforts.

### V. Collating, summarising and reporting the results

The data were collated, summarised and a narrative report of the findings was carried out in relation to the study’s purpose, the implications for future research, policy, collaborations, and practice.

## Results

### Publications included for analysis

**Fig 2:**
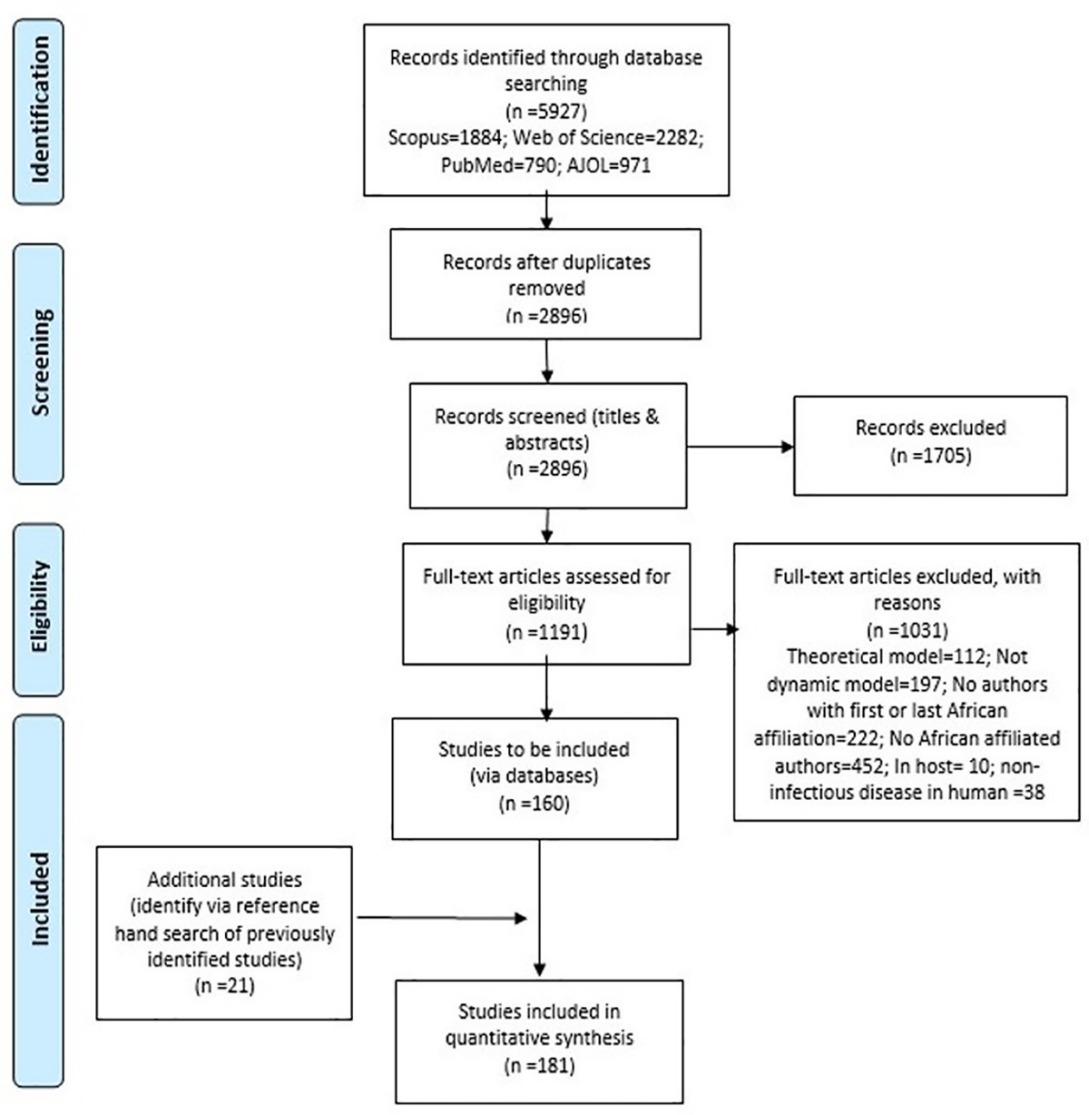
Flow chart illustrating the identification, screening, eligibility, and inclusion of literature for the scoping review process.

Of 5927 articles identified through the four specified databases (refer to Fig 2), 2896 publications were screened after removal of duplicates, and 1191 full-text publications were assessed for eligibility using the inclusion criteria and screening algorithm. Only 181 of these publications met the inclusion criteria for the study. The first phase of screening resulted in 160 publications, while an additional 21 articles were obtained from the reference hand search of the previously identified included studies (refer to Fig 2). Other studies were excluded since they involved infectious diseases in non-humans (n = 38), theoretical models (n = 112), non-dynamic models (n = 197), had no first or last author with an African institutional affiliation (n = 222), no African institutional affiliated authors (n = 452), or focused on in-host models (n = 10).

### African institutional authorship

The review identified 143 publications with African institutional affiliated first authors and 81 with both first and last authors AIA (S1 Table). Twenty-two of the African first authors were affiliated to at least two or more African institutions. Among the last authors, 18 had two African institutional affiliations, while three authors had three institutional affiliations.

### Applied epidemiological modelling research and authorship per disease

Table 1 shows some of the characteristics of the included articles. Most of the included articles focused on HIV (n = 55), malaria (n = 29), TB (n = 17) and Ebola virus disease (n = 8). The bulk of the research works were published by scientific journals such as *PLoS ONE*, *PLoS Neglected Tropical Diseases* and *AIDS* (Table 1 and S1 Table).

**Table 1:** The characteristics of the included articles.

Categories		Counts
		
**Date of publication**		
	1961–1970	1
	1971–1980	1
	1981–1990	2
	1991–2000	9
	2001–2010	39
	2011–2020	128
		
**Infectious diseases (first ten)**		
	HIV	55
	Malaria	29
	Tuberculosis	17
	Ebola virus disease	8
	Cholera	7
	Trypanosomiasis	7
	Pneumococcal infection	6
	Schistosomiasis	5
	STI	4
	Visceral leishmaniasis	4
		
**Peer-reviewed journals (first ten)**		
	PLoS ONE	15
	PLoS Neglected Tropical Diseases	8
	AIDS	8
	Malaria Journal	6
	Mathematical Biosciences	5
	Journal of Acquired Immune Deficiency Syndromes	5
	Infectious Disease Modelling	4
	Computational and Mathematical Methods in Medicine	4
	Sexually Transmitted Infections	4
	Vaccine	4

### Temporal publication trends

**Fig 3:**
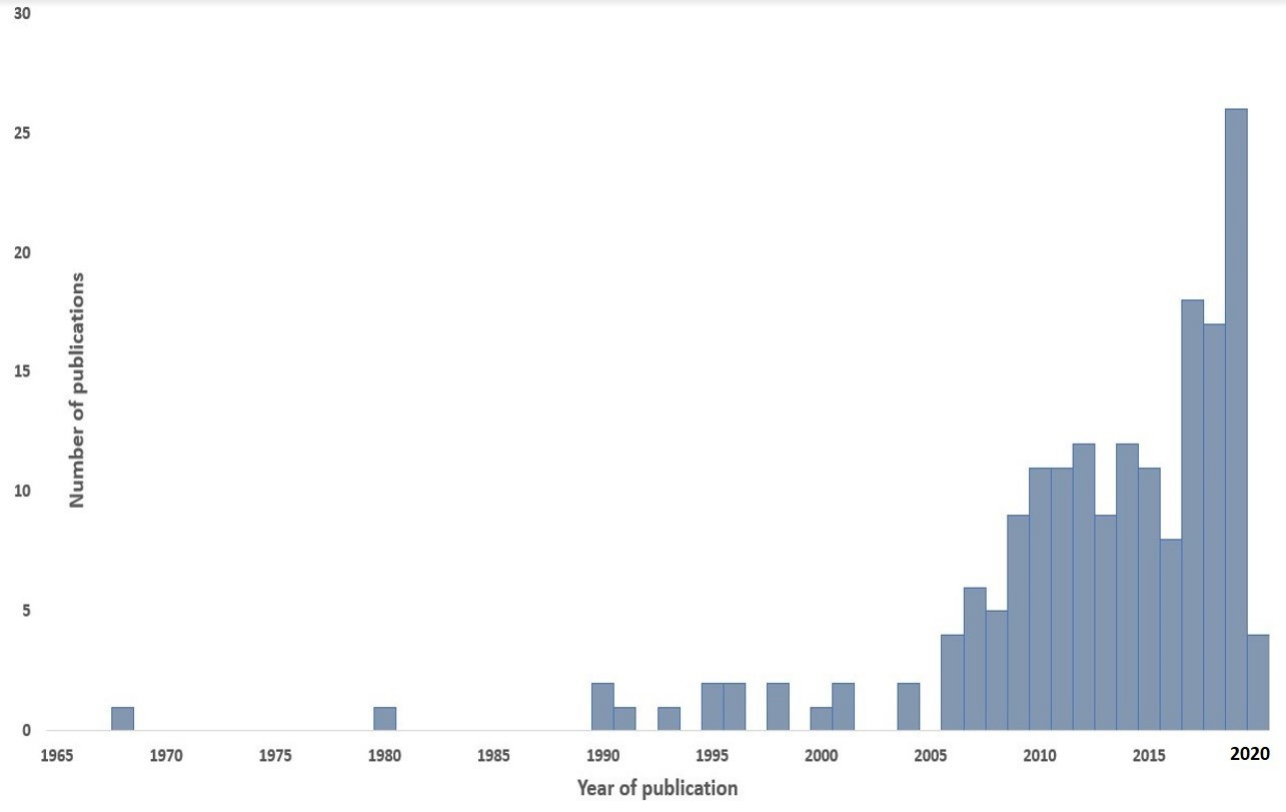
The number of publications on applied epidemiological modelling of infectious diseases with first and last African institutional affiliated authors by year of publication.

There has been a progressive increase year on year in the number of peer-reviewed publications with either the first or last authors being AIA (Fig 3). Applied epidemiological modelling studies on infectious diseases by AIA authors were scarce and sporadic until the last fifteen years. Sixty-eight percent of the included studies were published between 2011 and 2020.

### Applied epidemiological modelling hotspots and collaborations

**Fig 4:**
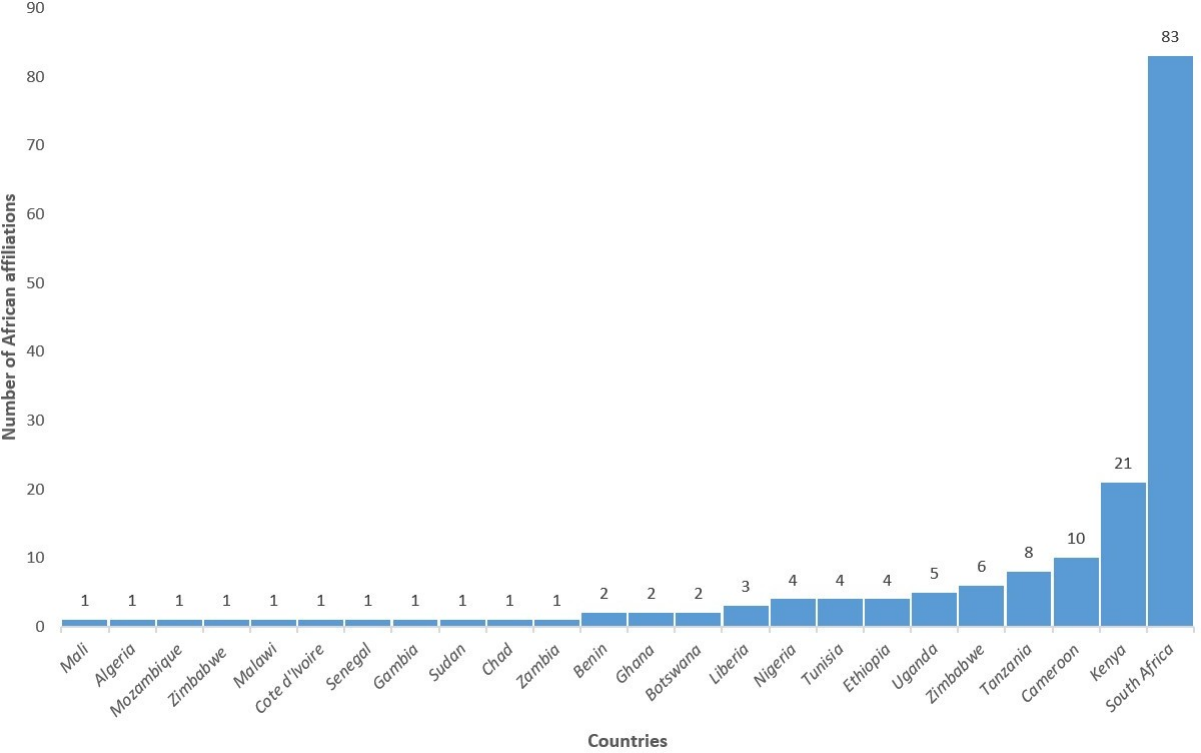
The number of publications with African institutional affiliated first authors by country of affiliation.

**Fig 5:**
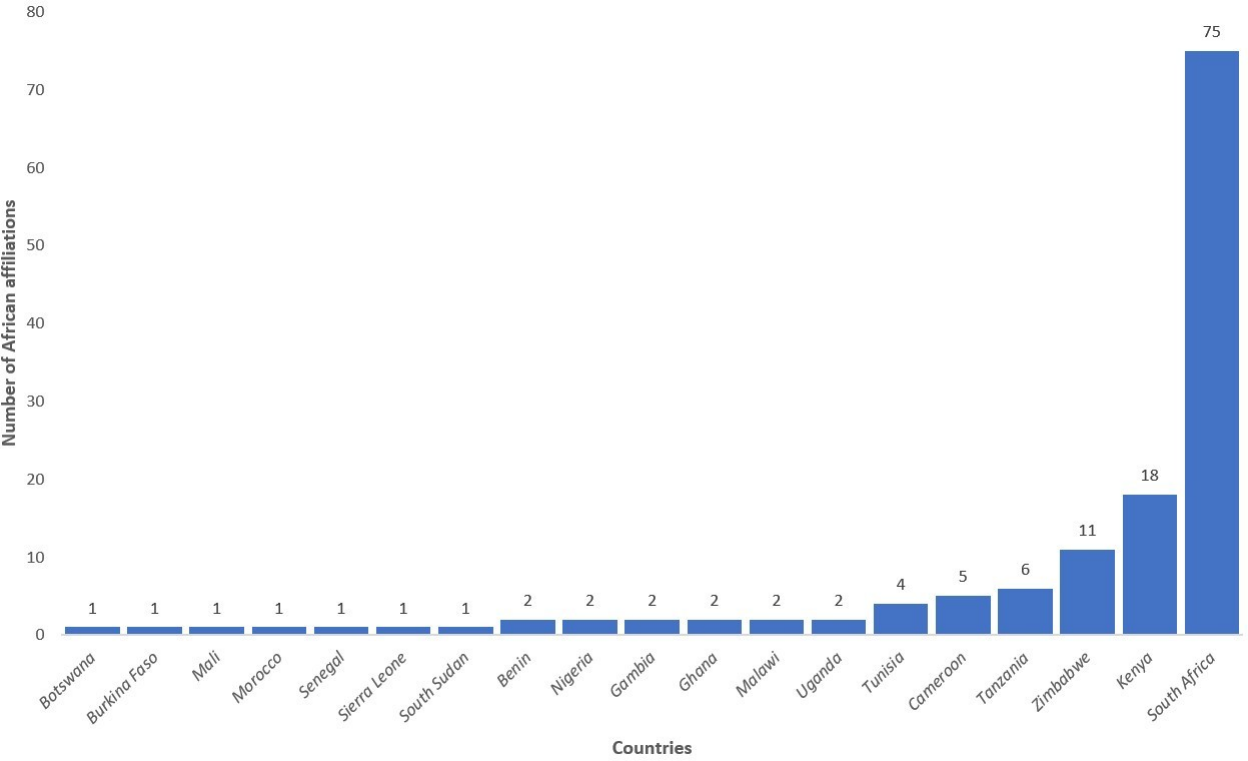
The number of publications with African institutional affiliated last authors by country of affiliation.

South African research institutions accounted for most of the first and last authors’ affiliations (Figs 4 and 5). Eighty-three of the publications had a South African first author. Research institutions such as the Centre for Infectious Disease Epidemiology and Research (CIDER) at the University of Cape Town, and DSI-NRF Centre of Excellence in Epidemiological Modelling and Analysis (SACEMA) hosted by Stellenbosch University, contributed to most of the research work. Researchers from these two institutions accounted for 15 and 10 of the included publications as first authors, respectively. Other institutions in South Africa, Kenya, Tanzania, and Zimbabwe also made substantial contributions (S2 Table). S2 Table also shows the order of listing of first and last authors with various African institutions’ affiliations. We identified 51 universities out of the 1225 recognized universities in Africa [18], while the other 56 institutions are research institutes and private organizations.

**Fig 6:**
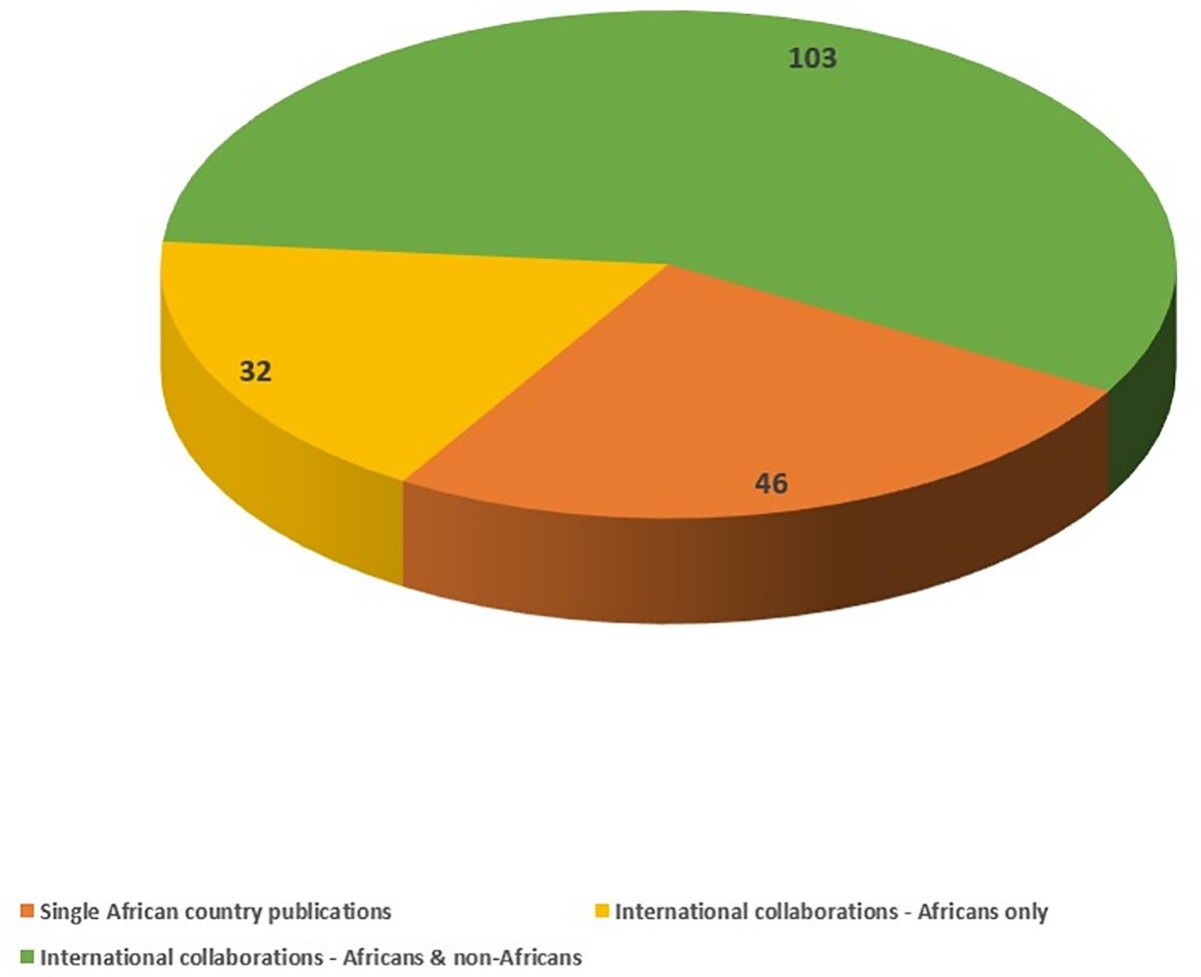
The number of collaborative publications between African and non-African authors.

Forty-six (25%) of the included studies were conducted solely by researchers that were based in a single African country while 32 (18%) were as a result of Africa-only international collaborations. One hundred and three (57%) studies were international collaborations involving both African and non-African researchers (Fig 6). Researchers from the United Kingdom and the United States accounted for 26% and 28% of the non-African collaborations, respectively.

## Discussion and recommendations

Peer-reviewed journal authorship of applied epidemiological modelling research on infectious diseases among African affiliated researchers was appraised for this study. HIV, malaria, TB, and Ebola virus disease were found to be the most researched infectious diseases in Africa. Furthermore, in the last six decades (1961–2020), the number of infectious disease modelling studies conducted by first or last AIA authors has gradually increased. Some publications with African affiliated researchers were excluded from the quantitative analysis since the researchers were not the first or last authors. Additionally, a significant number of studies using African data were also identified; however, most did not involve any AIA authors. All of the latter indicates that while there is substantially more international collaborative activity in the field, in many of these collaborations, African affiliated researchers are either not playing a primary role or not getting recognition for playing a primary role and are under-represented.

Authorship positioning is a means of assessing the author’s involvement and leadership in the research conducted, acting as a measure of the ability of researchers in project management and decision-making in collaborative research [19]. The findings of this study reveal that applied epidemiological modelling research on infectious diseases conducted in Africa do not frequently feature AIA researchers as the lead authors. A report from the World Bank and Elsevier shows that research in Africa was mostly produced via international collaborations, and most of the studies conducted across Africa were mostly led by non-African researchers [20]. Furthermore, this study demonstrates many collaborative activities between research institutions in the western hemisphere and those in Africa.

Several studies have reported under-representation of African affiliated authors in different fields of health-related research. More specifically, Mbaye *et al*. assessed the contribution of African researchers on six infectious diseases namely, HIV, malaria, TB, salmonellosis, Ebola viral disease and Buruli ulcer disease between 1980 and 2016 [19]. The study shows that most of the African first and last authors were affiliated to an institution in an Anglophone country, and most of the studies were extramurally funded with less than 10% African organizational funding [19]. Iyer’s work on authorship trends in *The Lancet Global Health* reveals under-representation and missed opportunities concerning global health research contributions by African researchers [21]. Of all the articles considered by Iyer, most of them focused on sub-Saharan Africa (40%); however, only 35% of the authors were affiliated with low-and-middle-income countries [21]. The findings by Mbaye and Iyer supported the findings of a prior survey that shows critical under-representation of developing country authors concerning biomedical publications in five high impact journals [22]. There were a significant number of articles with data from the developing countries but without any author affiliated with research institutions in the developing countries [23]. A systematic mapping of authorship on maternal health interventional research in low- and middle-income countries shows that only 54.5% of sub-Saharan African authors led studies in the region [22].

The total number of infectious disease modelling publications led by an AIA increased over the years with a similar pattern demonstrated by other related reviews [11, 21]. The increased volume of publications does imply likely increased capability in the institutions to conduct and convey study findings [21]; however, an increase in research output varies significantly between African countries [11, 21]. Public health and epidemiological research outputs varied between African countries with South Africa, Kenya, and Nigeria comprising about 40% of the overall publications between 1991 and 2010 [11].

Various reasons have been deduced for the observed under-representation of African affiliated researchers and these include lack or insufficient research funding, limited infrastructures, inadequate technical support, and insufficient training [22, 24]. Barriers such as donors’ conflicting agendas, competing commitments, fear of publication rejection, and language difficulty are some of the challenges facing the authors from developing countries [25]. There is a need for editors of scientific journals to be conversant with the real and apparent hurdles faced by researchers in developing countries [26]. Editors, researchers, and editorial organizations need to work collaboratively to bridge the north-south gap in scientific publication [26].

South Africa and Kenya emerged as the geographical hotspots for applied epidemiological modelling of infectious diseases in Africa. Factors like better funding and enhanced north-south collaboration relative to most of the other African countries could explain the observed trend [11]. For malaria research in Africa, countries like Kenya, Tanzania, Uganda, Malawi, and Ghana received substantial research investments. In contrast, other high burden countries like Sierra Leone, Guinea, and Central African Republic received little or no investments [27]. Countries with high funding for malaria control in Africa were also the ones with high research investments [27]. This shows inequitable research funding and operational investments across Africa. In South Africa, about 75% of the total health research expenditure originated from foreign sources, which highlighting increasing dependence on overseas funding. The South African government could also not meet all her commitment towards health research [28]. Nachega et al. suggested that funding for key research institutions, universities, and centres of excellence through governmental and other funders might have contributed better research productivity in African countries such as South Africa and Kenya [11]. Most of the other African research institutions are underfunded and get negligible funding for scientific research from government or local organizations [11]. This study shows some collaborations among researchers based in various African countries, while the south-south partnerships between African researchers and non-western ones were scanty.

HIV, malaria, and TB were demonstrated as the most researched infectious diseases by African researchers. Other studies on infectious diseases in Africa also revealed that these three infectious diseases were the most investigated in Africa in the last two decades [11, 17]. The 2016 Global Burden of Disease Study shows that HIV and malaria were the two key causes of years of life lost in sub-Saharan Africa [29]. HIV, malaria, and TB have further affected many people in Africa over the last thirty years [30–32]. More than 24.5 million people are currently living with HIV within the sub-Saharan African region, while malaria is endemic in most of the African countries [31, 32]. Conducting modelling and analysis of these infectious diseases is essential for monitoring transmission and assessing the effectiveness of intervention programmes. Robust research productivity, especially regarding African-based researchers’ involvement, is critical in developing policies that have local context, especially for common infectious diseases, emerging diseases of interest, epidemics, and pandemics.

This study is not without its limitations. Firstly, the authorship position may not reflect the contribution of the participating researchers [33, 34]. The last author may not necessarily be the senior author within the research team or may even be the least contributor to the work and not crucial in decision making for the research [33–35]. Secondly, many authors from Africa published their work while based in institutions outside Africa, and as such were classified as non-African. Similarly, this may also occur with a non-African researcher who had an African affiliation and was declared an African for this study. Thirdly, restricting this study to just English language articles also created room for the dropping of non-English language studies by African lead authors. Fourthly, restriction of authors’ affiliations to just two or three by scientific journals might have led to the non-inclusion of some African institutions by the lead authors. Lastly, many African affiliated researchers were found to concentrate on theoretical models, models involving non-humans, in-host models, and non-infectious disease models. Excluding these categories of models, could have led to the under-representation of overall modelling expertise in Africa.

Long-term north-south collaborations will aid in enhancing the capacity development of African scientists and enable wider dissemination of completed work via joint publications [11, 36]. Nachega *et al*. show that increasing north-south collaborations contributed to the rapid acceleration in research productivity in South Africa, Nigeria, Kenya, and Tanzania [11]. Collaborative research should further be linked to postgraduate training programmes in infectious disease modelling and epidemiology, thereby establishing key opportunities for pragmatic capacity development of high-quality infectious disease modelling experts in Africa [11, 37]. To increase infectious diseases modelling research outputs in Africa, there is a need to support existing training institutions and centres of excellence. There should be an emphasis on postgraduate and postdoctoral level training opportunities in academia for Africa to be self-reliant in applied epidemiological modelling research and education. Public and private local investment in research and development will allow for the implementation of local research agenda and offers good motivation for safeguarding local dissemination and usage of research findings [38–40]. African researchers and institutions need to aim for further visibility beyond the region by publishing their works in journals recognised in indexed databases [19].

## Conclusions

Applied epidemiological modelling studies on infectious diseases has gradually increased in the region and, with several collaborative efforts within Africa and beyond. A substantial number of African affiliated authors took part in several studies but were not in either first or last authorship positions. Most of the African institutional affiliated authors were associated with institutions based in South Africa and Kenya. Additionally, HIV, malaria and TB were the most researched infectious diseases within the region. However, it is paramount to develop more research capacity by supporting existing institutions in Africa, encourage more collaborative efforts, and promote more public and private local funding that will address local health priorities and research agendas.

## Supporting information

S1 Checklist PRISMA-SrC checklist. (DOCX)

S1 Table Included publications for the scoping review. (DOCX)

S2 Table List of African institutional affiliations and count. (DOCX)
