# Supplementary material for: African based researchers’ output on models for the transmission dynamics of infectious diseases and public health interventions: A scoping review
Source: PLoS One. 2021 May 6;16(5):e0250086. doi: 10.1371/journal.pone.0250086 (PMC8101744; doi:10.1371/journal.pone.0250086)
Supplement: S1 Table — (DOCX) [file pone.0250086.s002.docx]

| S1 Table. Included publications for the scoping review | | | | |  |
| --- | --- | --- | --- | --- | --- |
| Author | **Year** | **Journal** | **Infectious disease** | **Collaboration with other African researchers** | **Collaboration with non-African institutions** |
| Abakar *et al.* [1] | 2017 | PLoS Neglected Tropical Diseases | Tuberculosis | yes | yes |
| Abiodun *et al.* [2] | 2019 | International Journal of Environmental Research and Public Health | Malaria | yes | yes |
| Abiodun *et al*. [3] | 2019 | Malaria Journal | Malaria | no | no |
| Abiodun *et al.* [4] | 2018 | Hacettepe Journal of Mathematics and Statistics | Malaria | no | no |
| Abiodun *et al.* [5] | 2018 | The Open Infectious Diseases Journal | Malaria | no | no |
| Ackley *et al.* [6] | 2017 | PLoS Neglected Tropical Diseases | Trypanosomiasis | no | yes |
| Adam *et al.* [7] | 2009 | South African Medical Journal | HIV | no | no |
| Anderson *et al.* [8] | 2019 | PLoS Neglected Tropical Diseases | Rabies | no | yes |
| Andrew *et al.* [9] | 2013 | American Journal of Epidemiology | Tuberculosis | no | yes |
| Andrew *et al.* [10] | 2014 | The Journal of Infectious Diseases | Tuberculosis | no | yes |
| Anglaret *et al.* [11] | 2013 | Antiviral Therapy | HIV | no | yes |
| Asamoah *et al.* [12] | 2018 | Computational and Mathematical Methods in Medicine | Bacterial meningitis | yes | yes |
| Assana *et al.* [13] | 2019 | Preventive Veterinary Medicine | Taeniasis | no | no |
| Atashili *et al.* [14] | 2008 | BMC Infectious Diseases | HIV | no | yes |
| Bacaër *et al.* [15] | 2006 | Mathematical Biology | Cutaneous leishmaniasis | no | yes |
| Bakare *et al.* [16] | 2015 | International Journal of Ecological Economics and Statistics | Malaria | no | no |
| Baryarama *et al.* [17] | 2006 | Computational and Mathematical Methods in Medicine | HIV | no | no |
| Basu *et al.* [18] | 2007 | The Lancet | Tuberculosis | no | yes |
| Basu et al. [19] | 2009 | Proceedings of the Nat. Acad of Sciences of the United States of America | Tuberculosis | no | yes |
| Berge *et al.* [20] | 2017 | Journal of Biological Dynamics | Ebola virus disease | yes | no |
| Beyene *et al.* [21] | 2019 | One Health | Rabies | no | yes |
| Blaser *et al*. [22] | 2016 | Epidemics | Tuberculosis | yes | yes |
| Bowong *et al.* [23] | 2010 | International Journal of Bifurcation and Chaos | Tuberculosis | no | yes |
| Bowong *et al.* [24] | 2012 | Nonlinear Dynamics | Tuberculosis | no | yes |
| Bradley *et al.* 25] | 1996 | Transactions of the Royal Society of Tropical Medicine and Hygiene | Cholera | no | yes |
| Briscoe *et al.* [26] | 1980 | International Journal of Epidemiology | Communicable diseases | no | no |
| Cancre *et al.* [27] | 2000 | American Journal of Epidemiology | Malaria | no | yes |
| Cheke *et al*. [28] | 2015 | Philosophical Transactions of the Royal Society B | Onchocerciasis | no | yes |
| Chidumayo *et al.* [29] | 2018 | PLoS ONE | Rabies | no | no |
| Chigidi *et al.* [30] | 2009 | Mathematical Biosciences and Engineering | HIV | yes | no |
| Childs *et al.* [31] | 2010 | Mathematical Biosciences | Trypanosomiasis | no | no |
| Chirwa *et al.* [32] | 2013 | Global Health Action | Leprosy | no | yes |
| Chiu *et al.* [33] | 2017 | BMC Public Health | HIV | no | yes |
| Chlif *et al.* [34] | 2016 | Eastern Mediterranean Health Journal | Influenza | no | no |
| Coffeng *et al.* [35] | 2013 | PLoS Neglected Tropical Diseases | Onchocerciasis | yes | yes |
| Collins *et al.* [36] | 2018 | Journal of Applied Mathematics | Cholera | no | no |
| Collins *et al.* [37] | 2014 | Journal of Theoretical Biology | Cholera | yes | no |
| Cowan *et al.* [38] | 2019 | Journal of the International AIDS Society | HIV | no | yes |
| Dangbe *et al.* [39] | 2018 | Mathematical Biosciences | Cholera | no | yes |
| Desmond *et al.* [40] | 2020 | AIDS Care | HIV | no | yes |
| Djidjou-Demasse *et al.* [41] | 2019 | Studies in Applied Mathematics | Malaria | no | yes |
| Dodd *et al.* [42] | 2011 | PLoS ONE | Tuberculosis | no | yes |
| Dube *et al.* [43] | 2008 | Journal of Acquired Immune Deficiency Syndromes | HIV | no | yes |
| Dye *et al.* [44] | 1998 | The Lancet | Tuberculosis | no | yes |
| Elmojtaba *et al.* [45] | 2010 | Applied Mathematics and Computation | Visceral leishmaniasis | yes | no |
| Fallah *et al.* [46] | 2015 | PLoS Neglected Tropical Diseases | Ebola virus disease | no | yes |
| Fasina *et al.* [47] | 2014 | Euro Surveillance | Ebola virus disease | yes | yes |
| Flasche *et al.* [48] | 2017 | BMC Medicine | Pneumococcal infection | no | yes |
| Fryer *et al.* [49] | 2015 | AIDS | HIV | yes | yes |
| Gashaw *et al.* [50] | 2019 | International Journal of Biomathematics | Malaria | yes | no |
| Gaudart *et al.* [51] | 2009 | Malaria Journal | Malaria | no | yes |
| Getz *et al.* [52] | 2015 | Computational and Mathematical Methods in Medicine | Ebola virus disease | yes | yes |
| Glynn *et al.* [53] | 2003 | Journal of Acquired Immune Deficiency Syndromes | HIV | yes | yes |
| Godana *et al.* [54] | 2019 | PLoS ONE | Visceral leishmaniasis | no | no |
| Guwatudde *et al*. [55] | 2004 | Preventive Medicine | Tuberculosis | no | yes |
| Hallett *et al.* [56] | 2010 | Bulletin of the World Health Organization | HIV | no | yes |
| Hargrove *et al.* [57] | 1998 | Bulletin of Entomological Research | Trypanosomiasis | yes | no |
| Hargrove *et al.* [58] | 2012 | PLoS Neglected Tropical Diseases | Trypanosomiasis | no | yes |
| Hassan *et al.* [59] | 2014 | Computational and Mathematical Methods in Medicine | Tuberculosis | no | yes |
| Hay *et al.* [60] | 2001 | Trends in Parasitology | Malaria | no | yes |
| Heidenberger *et al* [61] | 1993 | European Journal of Operational Research | HIV | no | yes |
| Hippner *et al.* [62] | 2019 | PLoS ONE | Tuberculosis | no | yes |
| Hontelez *et al.* [63] | 2013 | PLoS Medicine | HIV | no | yes |
| Hontelez *et al.* [64] | 2012 | AIDS | HIV | no | yes |
| Hontelez *et al.* [65] | 2011 | PLoS ONE | HIV | no | yes |
| Hontelez *et al.* [66] | 2011 | Vaccine | Rotavirus | no | yes |
| Hove-Musekwa *et al.* [67] | 2011 | Bulletin of Mathematical Biology | HIV | yes | no |
| Hussaini *et al.* [68] | 2017 | Infectious Disease Modelling | Visceral leishmaniasis | no | yes |
| Isdory *et al.* [69] | 2015 | PLoS ONE | HIV | no | yes |
| Johnson *et al.* [70] | 2015 | AIDS | HIV | no | yes |
| Johnson *et al.* [71] | 2017 | PLoS Medicine | HIV | no | yes |
| Johnson *et al.* [72] | 2012 | The Pediatric Infectious Disease Journal | HIV | no | yes |
| Johnson *et al.* [73] | 2019 | Scientific Reports | HIV | no | no |
| Johnson *et al.* [74] | 2007 | Vaccine | HIV | no | no |
| Johnson *et al.* [75] | 2012 | Journal of the Royal Society Interface | HIV | no | yes |
| Johnson *et al.* [76] | 2016 | Global Health Action | HIV | no | no |
| Johnson *et al.* [77] | 2012 | Journal of Acquired Immune Deficiency Syndromes | HIV | no | yes |
| Johnson *et al.* [78] | 2017 | Southern African Journal of HIV Medicine | HIV | no | no |
| Johnson *et al.* [79] | 2011 | Sexual & Reproductive Healthcare | HIV | no | no |
| Johnson *et al.* [80] | 2016 | Sexually Transmitted Infections | HIV | no | yes |
| Johnson *et al.* [81] | 2007 | South African Journal of Science | HIV | no | no |
| Johnson *et al.* [82] | 2009 | Demographic Research | HIV | no | no |
| Johnson *et al.* [83] | 2006 | Demographic Research | HIV | no | no |
| Johnson *et al.* [84] | 2017 | Southern African Journal of HIV Medicine | STI | no | no |
| Johnson *et al.* [85] | 2011 | Epidemiology and Infection | STI | no | no |
| Johnson *et al.* [86] | 2012 | Southern African Journal of HIV Medicine | STI | no | no |
| Johnson *et al.* [87] | 2012 | Tropical Medicine and International Health | STI | no | no |
| Johnson *et al.* [88] | 2009 | Sexually Transmitted Infections | STI | no | yes |
| Kaabi *et al.* [89] | 2013 | BioSystems | Cutaneous leishmaniasis | no | no |
| Kaabiet *et al.* [90] | 2018 | Acta Tropica | Visceral leishmaniasis | no | no |
| Kajunguri *et al.* [91] | 2014 | Bulletin of Mathematical Biology | Trypanosomiasis | yes | yes |
| Kakmeni *et al.* [92] | 2018 | International Journal of Health Geographics | Malaria | yes | no |
| Kalinda *et al*. [93] | 2019 | BioSystems | Schistosomiasis | no | yes |
| Kendall *et al.* [94] | 2019 | AIDS | HIV/TB | no | yes |
| Killeen *et al.* [95] | 2007 | PLoS Medicine | Malaria | no | yes |
| Killeen *et al.* [96] | 2007 | Transactions of the Royal Society of Tropical Medicine and Hygiene | Malaria | no | yes |
| Killeen *et al.* [97] | 2006 | The American Society of Tropical Medicine and Hygiene | Malaria | no | yes |
| Killeen *et al.* [98] | 2001 | Transactions of the Royal Society of Tropical Medicine and Hygiene | Malaria | no | yes |
| Kimani *et al.* [99] | 2016 | EcoHealth | Rift valley fever | no | yes |
| Kinyanjui *et al.* [100] | 2020 | Vaccine: X | RSV infection | no | yes |
| Kivuti-Bitok *et al.* [101] | 2014 | Cost Effectiveness and Resource Allocation | HPV infection | no | yes |
| Kiware *et al.* [102] | 2012 | PLoS ONE | Malaria | no | yes |
| Kokomo *et al*. [103] | 2020 | Nonlinear Analysis: RealWorld Applications | Cholera | yes | no |
| Kombe *et al.* [104] | 2019 | Epidemics | RSV infection | no | yes |
| Landouré *et al*. [105] | 2012 | PLoS Neglected Tropical Diseases | Schistosomiasis | yes | yes |
| Lekone *et al*. [106] | 2008 | Biometrical Journal | Severe Acute Respiratory Syndrome | no | no |
| Levy *et al*. [107] | 2017 | Infectious Disease Modelling | Ebola virus disease | yes | yes |
| Lilian *et al.* [108] | 2014 | Journal of Acquired Immune Deficiency Syndromes | HIV | no | no |
| Lopman *et al*. [109] | 2009 | Sexually Transmitted Diseases | HIV | no | yes |
| Lopman *et al*. [110] | 2008 | PLoS ONE | HIV | no | yes |
| Losio *et al*. [111] | 2018 | Journal of Applied Mathematics | Dracontiasis | yes | no |
| Lourenço *et al*. [112] | 2019 | BMC Medicine | Pneumococcal infection | no | yes |
| Macdonald *et al*. [113] | 1968 | Bulletin of the World Health Organization | Malaria | no | yes |
| Mahiane *et al.* [114] | 2014 | Statistics in Medicine | HIV | no | yes |
| Mahiane *et al*. [115] | 2010 | Journal of the Royal Statistical Society | HIV/HSV-2 | yes | yes |
| Maleta *et al*. [116] | 2010 | BMC Health Services Research | HIV | no | no |
| Maliyoni *et al*. [117] | 2017 | Bulletin of Mathematical Biology | Tick-borne disease | no | yes |
| Malunguza *et al*. [118] | 2017 | Journal of Theoretical Biology | HIV | no | yes |
| Marx *et al*. [119] | 2018 | The Lancet Global Health | Tuberculosis | no | yes |
| Mengistu *et al*. [120] | 2019 | Journal of Applied Mathematics | Tuberculosis | no | no |
| Mnyone *et al*. [121] | 2012 | Malaria Journal | Malaria | no | yes |
| Moiroux *et al*. [122] | 2014 | PLoS ONE | Malaria | yes | yes |
| Mukhtar *et al*. [123] | 2018 | PLoS ONE | Malaria | no | no |
| Mukhtar *et al.* [124] | 2019 | Mathematical Biosciences | Malaria | no | no |
| Mukhtar *et al*. [125] | 2019 | Mathematical Biosciences | Malaria | no | no |
| Muller *et al*. [126] | 2004 | Biological Modelling | Trypanosomiasis | no | yes |
| Musa *et al.* [127] | 2020 | Journal of Theoretical Biology | Lassa Fever | no | yes |
| Mwasa *et al*. [128] | 2011 | BioSystems | Cholera | yes | no |
| Mwesigwa *et al.* [129] | 2019 | Malaria Journal | Malaria | no | yes |
| Nadhem *et al*. [130] | 2015 | Health Economics Review | Ebola virus disease | no | no |
| Nagelkerke *et al.* [131] | 1990 | Infectious Disease Modelling | HIV | no | yes |
| Nagelkerke *et al.* [132] | 2018 | AIDS | HIV | no | yes |
| Nagelkerke *et al.* [133] | 1995 | Journal of Acquired Immune Deficiency Syndromes | HIV | no | yes |
| Nagelkerke *et al.* [134] | 2009 | AIDS | HIV | no | yes |
| Nannyonga *et al.* [135] | 2012 | PLoS ONE | Hepatitis E infection | no | yes |
| Ngarakana-Gwasira *et al*. [136] | 2016 | Malaria Research and Treatment | Malaria | no | no |
| Nguefack *et al.* [137] | 2016 | BMC Infectious Diseases | HIV | yes | yes |
| Nyabadza *et al.* [138] | 2011 | Journal of Theoretical Biology﻿ | Buruli ulcer | yes | no |
| Nyabadza *et al.* [139] | 2015 | BMC Research Notes | HIV | yes | yes |
| Nyabadza *et al.* [140] | 2011 | Nonlinear Analysis: Real World Applications | HIV | no | yes |
| Nyabadza *et al.* [141] | 2013 | South African Journal of Science | Tuberculosis | no | no |
| Ojal *et al.* [142] | 2017 | Vaccine | Pneumococcal infection | no | yes |
| Ojal *et al*. [143] | 2019 | The Lancet Global Health | Pneumococcal infection | no | yes |
| Okango *et al*. [144] | 2016 | BMC Public Health | HIV/HSV-2 | yes | no |
| Okumu *et al*. [145] | 2010 | PLoS ONE | Malaria | no | yes |
| Omondi *et al.* [146] | 2017 | Cogent Mathematics & Statistics | HIV | yes | no |
| Omondi *et al.* [147] | 2019 | Infectious Disease Modelling | HIV | no | no |
| Omondi *et al*. [148] | 2018 | International Journal of Applied and Computational Mathematics | HIV | no | no |
| Omondi *et al.* [149] | 2018 | Letters in Biomathematics | HIV | no | no |
| Omondi *et al.* [150] | 2018 | Mathematical Biosciences | Onchocerciasis | no | no |
| Otiende *et al*. [151] | 2019 | BMC Infectious Diseases | HIV/TB | yes | yes |
| Pearson *et al.* [152] | 2019 | Journal of the Royal Society Interface | Dengue Fever | no | yes |
| Pretorius *et al.* [153] | 2010 | PLoS ONE | HIV | no | yes |
| Qureshi *et al*. [154] | 2019 | Physica A | Dengue Fever | no | yes |
| Sartorius *et al*. [155] | 2013 | Bulletin of the World Health Organization | Measles | no | no |
| Shiri *et al*. [156] | 2013 | American Journal of Epidemiology | Pneumococcal infection | no | yes |
| Silal *et al.* [157] | 2014 | Malaria Journal | Malaria | no | yes |
| Silal *et al.* [158] | 2015 | Malaria Journal | Malaria | no | yes |
| Silhol *et al.* [159] | 2017 | AIDS | HIV | no | yes |
| Ssebuliba *et al.* [160] | 2017 | Epidemiology and Infection | Malaria/Pneumonia | yes | yes |
| Steen *et al.* [161] | 2014 | AIDS | HIV | no | yes |
| Sumaye *et al.* [162] | 2019 | PLoS ONE | Rift valley fever | no | yes |
| Torr *et al.* [163] | 2011 | PLoS Neglected Tropical Diseases | Trypanosomiasis | no | yes |
| Tulu *et al*. [164] | 2017 | Advances in Difference Equations | Ebola virus disease | no | yes |
| Tulu *et al.* [165] | 2017 | Results in Physics | Ebola virus disease | no | yes |
| van den Berghy *et al*. [166] | 2008 | Orion | Cholera | no | no |
| van Schalkwyk *et al.* [167] | 2018 | Sexually Transmitted Infections | HIV/HPV | no | no |
| van Schalkwyk *et al.* [168] | 2019 | Vaccine | HPV infection | no | no |
| Vandormael *et al.* [169] | 2018 | International Journal of Epidemiology | HIV | no | yes |
| Vermeulen *et al.* [170] | 2019 | Vox Sanguinis | HTLV 1 & 2 infection | no | yes |
| Vickerman *et al.* [171] | 2010 | Sexually Transmitted Infections | HIV/STI | no | yes |
| Viljoen *et al.* [172] | 2014 | Acta Biotheoretica | HIV | no | yes |
| Williams *et al.* [173] | 1995 | Proceedings of the Nat. Acad. of Sciences of the United States of America | HIV/TB | no | yes |
| Williams *et al*. [174] | 2010 | Epidemiology and Infection | Measles | no | yes |
| Wood *et al.* [175] | 2010 | Clinical Infectious Diseases | Tuberculosis | no | yes |
| Woolhouse *et al*. [176] | 1990 | Parasitology | Schistosomiasis | no | yes |
| Woolhouse *et al*. [177] | 1991 | Philosophical Transactions of the Royal Society B | Schistosomiasis | no | yes |
| Woolhouse *et al.* [178] | 1996 | Tropical Medicine and International Health | Schistosomiasis | no | yes |
| Ye *et al.* [179] | 2007 | Annals of Tropical Medicine & Parasitology | Malaria | no | yes |
| Ye *et al.* [180] | 2009 | Global Health Action | Malaria | no | yes |
| Younsi *et al.* [181] | 2015 | Tsinghua Science and Technology | Influenza | no | yes |

**Abbreviations:** HIV- human immunodeficiency virus; HPV- human papillomavirus; HSV- herpes simplex virus; HTLV- Human T-lymphotropic virus; RSV- respiratory syncytial virus; STI- sexually transmitted infection; TB- tuberculosis

**References**

1. Abakar MF, Yahyaoui Azami H, Justus Bless P, Crump L, Lohmann P, Laager M, Chitnis N, Zinsstag J. Transmission dynamics and elimination potential of zoonotic tuberculosis in morocco. PLoS neglected tropical diseases. 2017 Feb 2;11(2):e0005214.
2. Abiodun GJ, Maharaj R, Witbooi P, Okosun KO. Modelling the influence of temperature and rainfall on the population dynamics of Anopheles arabiensis. Malaria journal. 2016 Dec 1;15(1):364.
3. Abiodun GJ, Makinde OS, Adeola AM, Njabo KY, Witbooi PJ, Djidjou-Demasse R, Botai JO. A dynamical and zero-inflated negative binomial regression modelling of malaria incidence in Limpopo Province, South Africa. International Journal of Environmental Research and Public Health. 2019 Jan;16(11):2000.
4. Abiodun GJ, Witbooi P, Okosun KO. Modelling the impact of climatic variables on malaria transmission. Hacettepe J. Math. Stat. 2018 Apr 1;47:219-35.
5. Abiodun GJ, Witbooi PJ, Okosun KO, Maharaj R. Exploring the impact of climate variability on malaria transmission using a dynamic mosquito-human malaria model. The open infectious diseases journal. 2018;10:88.
6. Ackley SF, Hargrove JW. A dynamic model for estimating adult female mortality from ovarian dissection data for the tsetse fly Glossina pallidipes Austen sampled in Zimbabwe. PLoS neglected tropical diseases. 2017 Aug 30;11(8):e0005813.
7. Adam MA, Johnson LF. Estimation of adult antiretroviral treatment coverage in South Africa. South African Medical Journal. 2009;99(9).
8. Anderson A, Kotzé J, Shwiff SA, Hatch B, Slootmaker C, Conan A, Knobel D, Nel LH. A bioeconomic model for the optimization of local canine rabies control. PLOS Neglected Tropical Diseases. 2019 May 22;13(5):e0007377.
9. Andrews JR, Morrow C, Walensky RP, Wood R. Integrating social contact and environmental data in evaluating tuberculosis transmission in a South African township. The Journal of infectious diseases. 2014 Aug 15;210(4):597-603.
10. Andrews JR, Morrow C, Wood R. Modeling the role of public transportation in sustaining tuberculosis transmission in South Africa. American journal of epidemiology. 2013 Mar 15;177(6):556-61.
11. Anglaret X, Scott CA, Walensky RP, Ouattara E, Losina E, Moh R, Becker JE, Uhler L, Danel C, Messou E, Eholié S. Could early antiretroviral therapy entail more risks than benefits in sub-Saharan African HIV-infected adults? A model-based analysis. Antiviral therapy. 2013;18(1):45.
12. Asamoah JK, Nyabadza F, Seidu B, Chand M, Dutta H. Mathematical modelling of bacterial meningitis transmission dynamics with control measures. Computational and mathematical methods in medicine. 2018 Jan 1;2018.
13. Assana E, Awah-Ndukum J, Djonmaïla JD, Zoli AP. Prevalence of porcine Taenia solium and Taenia hydatigena cysticercosis in Cameroon. Preventive veterinary medicine. 2019 Aug 1;169:104690.
14. Atashili J, Kalilani L, Seksaria V, Sickbert-Bennett EE. Potential impact of infant feeding recommendations on mortality and HIV-infection in children born to HIV-infected mothers in Africa: a simulation. BMC infectious diseases. 2008 Dec 1;8(1):66.
15. Bacaër N, Guernaoui S. The epidemic threshold of vector-borne diseases with seasonality. Journal of mathematical biology. 2006 Sep 1;53(3):421-36.
16. Bakare EA. On the Qualitative behaviour of a human-mosquito model for Malaria with multiple vector control strategies. International Journal of Ecological Economics and Statistics. 2015 Jan 1;36(1):96-113.
17. Baryarama F, Mugisha JY, Luboobi LS. A mathematical model for the dynamics of HIV/AIDS with gradual behaviour change. Computational and Mathematical Methods in Medicine. 2006 Mar 1;7(1):15-26.
18. Basu S, Andrews JR, Poolman EM, Gandhi NR, Shah NS, Moll A, Moodley P, Galvani AP, Friedland GH. Prevention of nosocomial transmission of extensively drug-resistant tuberculosis in rural South African district hospitals: an epidemiological modelling study. The Lancet. 2007 Oct 27;370(9597):1500-7.
19. Basu S, Friedland GH, Medlock J, Andrews JR, Shah NS, Gandhi NR, Moll A, Moodley P, Sturm AW, Galvani AP. Averting epidemics of extensively drug-resistant tuberculosis. Proceedings of the National Academy of Sciences. 2009 May 5;106(18):7672-7.
20. Berge T, Lubuma JS, Moremedi GM, Morris N, Kondera-Shava R. A simple mathematical model for Ebola in Africa. Journal of biological dynamics. 2017 Jan 1;11(1):42-74.
21. Beyene TJ, Fitzpatrick MC, Galvani AP, Mourits MC, Revie CW, Cernicchiaro N, Sanderson MW, Hogeveen H. Impact of One-Health framework on vaccination cost-effectiveness: A case study of rabies in Ethiopia. One Health. 2019 Dec 1;8:100103.
22. Blaser N, Zahnd C, Hermans S, Salazar-Vizcaya L, Estill J, Morrow C, Egger M, Keiser O, Wood R. Tuberculosis in Cape Town: an age-structured transmission model. Epidemics. 2016 Mar 1;14:54-61.
23. Bowong S, Kurths J. Modeling and parameter estimation of Tuberculosis with application to Cameroon. International Journal of Bifurcation and Chaos. 2011 Jul;21(07):1999-2015.
24. Bowong S, Kurths J. Modeling and analysis of the transmission dynamics of tuberculosis without and with seasonality. Nonlinear Dynamics. 2012 Feb 1;67(3):2027-51.
25. Bradley M, Shakespeare R, Ruwende A, Woolhouse ME, Mason E, Munatsi A. Epidemiological features of epidemic cholera (El Tor) in Zimbabwe. Transactions of the Royal Society of Tropical Medicine and Hygiene. 1996 Jul 1;90(4):378-82.
26. Briscoe J. On the use of simple analytic mathematical models of communicable diseases. International journal of epidemiology. 1980 Sep 1;9(3):265-70.
27. Cancre N, Tall A, Rogier C, Faye J, Sarr O, Trape JF, Spiegel A, Bois F. Bayesian analysis of an epidemiologic model of Plasmodium falciparum malaria infection in Ndiop, Senegal. American journal of epidemiology. 2000 Oct 15;152(8):760-70.
28. Cheke RA, Basánez MG, Perry M, White MT, Garms R, Obuobie E, Lamberton PH, Young S, Osei-Atweneboana MY, Intsiful J, Shen M. Potential effects of warmer worms and vectors on onchocerciasis transmission in West Africa. Philosophical Transactions of the Royal Society B: Biological Sciences. 2015 Apr 5;370(1665):20130559.
29. Chidumayo NN. System dynamics modelling approach to explore the effect of dog demography on rabies vaccination coverage in Africa. PloS one. 2018 Oct 25;13(10):e0205884.
30. Chigidi E, Lungu EM. HIV model incorporating differential progression for treatment-naive and treatment-experienced infectives. Mathematical Biosciences & Engineering. 2009 Jul 1;6(3):427.
31. Childs SJ. The finite element implementation of a KPP equation for the simulation of tsetse control measures in the vicinity of a game reserve. Mathematical biosciences. 2010 Sep 1;227(1):29-43.
32. Chirwa T, Floyd S, Fine P. Estimating the extent of household contact misclassification with index cases of disease in longitudinal studies using a stochastic simulation model. Global health action. 2013 Dec 1;6(1):19614.
33. Chiu C, Johnson LF, Jamieson L, Larson BA, Meyer-Rath G. Designing an optimal HIV programme for South Africa: Does the optimal package change when diminishing returns are considered?. BMC Public Health. 2017 Dec 1;17(1):143.
34. Chlif S, Aissi W, Bettaieb J, Kharroubi G, Nouira M, Yazidi R, El Moussi A, Slim L, Ben Salah A. Modelling of seasonal influenza and estimation of the burden in Tunisia. EMHJ-Eastern Mediterranean Health Journal. 2016;22(7):459-66.
35. Coffeng LE, Stolk WA, Zoure HG, Veerman JL, Agblewonu KB, Murdoch ME, Noma M, Fobi G, Richardus JH, Bundy DA, Habbema D. African Programme For Onchocerciasis Control 1995–2015: model-estimated health impact and cost. PLoS Negl Trop Dis. 2013 Jan 31;7(1):e2032.
36. Collins OC, Duffy KJ. Analysis and optimal control intervention strategies of a waterborne disease model: A realistic case study. Journal of Applied Mathematics. 2018 Nov 21;2018.
37. Collins OC, Govinder KS. Incorporating heterogeneity into the transmission dynamics of a waterborne disease model. Journal of theoretical biology. 2014 Sep 7;356:133-43.
38. Cowan FM, Chabata ST, Musemburi S, Fearon E, Davey C, Ndori‐Mharadze T, Bansi‐Matharu L, Cambiano V, Steen R, Busza J, Yekeye R. Strengthening the scale‐up and uptake of effective interventions for sex workers for population impact in Zimbabwe. Journal of the International AIDS Society. 2019 Jul;22:e25320.
39. Dangbé E, Irépran D, Perasso A, Békollé D. Mathematical modelling and numerical simulations of the influence of hygiene and seasons on the spread of cholera. Mathematical biosciences. 2018 Feb 1;296:60-70.
40. Desmond C, Labuschagne P, Cluver L, Tomlinson M, Richter L, Hunt X, Marlow M, Welte A. Modelling the impact of maternal HIV on uninfected children: correcting current estimates. AIDS care. 2020 Feb 7:1-9.
41. Djidjou‐Demasse R, Abiodun GJ, Adeola AM, Botai JO. Development and analysis of a malaria transmission mathematical model with seasonal mosquito life‐history traits. Studies in Applied Mathematics. 2020 May;144(4):389-411.
42. Dodd PJ, White RG, Corbett EL. Periodic active case finding for TB: when to look? PLoS One. 2011 Dec 22;6(12):e29130.
43. Dube S, Boily MC, Mugurungi O, Mahomva A, Chikhata F, Gregson S. Estimating vertically acquired HIV infections and the impact of the prevention of mother-to-child transmission program in Zimbabwe: insights from decision analysis models. JAIDS Journal of Acquired Immune Deficiency Syndromes. 2008 May 1;48(1):72-81.
44. Dye C, Garnett GP, Sleeman K, Williams BG. Prospects for worldwide tuberculosis control under the WHO DOTS strategy. The Lancet. 1998 Dec 12;352(9144):1886-91.
45. ELmojtaba IM, Mugisha JY, Hashim MH. Mathematical analysis of the dynamics of visceral leishmaniasis in the Sudan. Applied Mathematics and Computation. 2010 Nov 15;217(6):2567-78.
46. Fallah MP, Skrip LA, Gertler S, Yamin D, Galvani AP. Quantifying poverty as a driver of Ebola transmission. PLoS neglected tropical diseases. 2015 Dec 31;9(12):e0004260.
47. Fasina FO, Shittu A, Lazarus D, Tomori O, Simonsen L, Viboud C, Chowell G. Transmission dynamics and control of Ebola virus disease outbreak in Nigeria, July to September 2014. Eurosurveillance. 2014 Oct 9;19(40):20920.
48. Flasche S, Ojal J, De Waroux OL, Otiende M, O’Brien KL, Kiti M, Nokes DJ, Edmunds WJ, Scott JA. Assessing the efficiency of catch-up campaigns for the introduction of pneumococcal conjugate vaccine: a modelling study based on data from PCV10 introduction in Kilifi, Kenya. BMC medicine. 2017 Dec 1;15(1):113.
49. Fryer HR, Van Tienen C, Van Der Loeff MS, Aaby P, Da Silva ZJ, Whittle H, Rowland-Jones SL, de Silva TI. Predicting the extinction of HIV-2 in rural Guinea-Bissau. AIDS (London, England). 2015 Nov 28;29(18):2479.
50. Gashaw KW, Kassa SM, Ouifki R. Climate-dependent malaria disease transmission model and its analysis. International Journal of Biomathematics. Vol. 12, No. 8 (2019) 1950087.
51. Gaudart J, Touré O, Dessay N, lassane Dicko A, Ranque S, Forest L, Demongeot J, Doumbo OK. Modelling malaria incidence with environmental dependency in a locality of Sudanese savannah area, Mali. Malaria journal. 2009 Dec 1;8(1):61.
52. Getz WM, Gonzalez JP, Salter R, Bangura J, Carlson C, Coomber M, Dougherty E, Kargbo D, Wolfe ND, Wauquier N. Tactics and strategies for managing Ebola outbreaks and the salience of immunization. Computational and mathematical methods in medicine. 2015 Jan 1;2015.
53. Glynn JR, Caraël M, Buvé A, Musonda RM, Kahindo M. HIV risk in relation to marriage in areas with high prevalence of HIV infection. Journal of acquired immune deficiency syndromes (1999). 2003 Aug;33(4):526-35.
54. Godana AA, Mwalili SM, Orwa GO. Dynamic spatiotemporal modeling of the infected rate of visceral leishmaniasis in human in an endemic area of Amhara regional state, Ethiopia. PloS one. 2019 Mar 1;14(3):e0212934.
55. Guwatudde D, Debanne SM, Diaz M, King C, Whalen CC. A re-examination of the potential impact of preventive therapy on the public health problem of tuberculosis in contemporary sub-Saharan Africa. Preventive medicine. 2004 Nov 1;39(5):1036-46.
56. Hallett TB, Gregson S, Kurwa F, Garnett GP, Dube S, Chawira G, Mason PR, Nyamukapa CA. Measuring and correcting biased child mortality statistics in countries with generalized epidemics of HIV infection. Bulletin of the World Health Organization. 2010;88:761-8.
57. Hargrove JW, Williams BG. Optimized simulation as an aid to modelling, with an application to the study of a population of tsetse flies, Glossina morsitans morsitans (Diptera: Glossinidae). Bulletin of entomological research. 1998 Aug 1;88(4):425-35.
58. Hargrove JW, Ouifki R, Kajunguri D, Vale GA, Torr SJ. Modeling the control of trypanosomiasis using trypanocides or insecticide-treated livestock. PLoS Negl Trop Dis. 2012 May 15;6(5):e1615.
59. Hassan AS, Garba SM, Gumel AB, Lubuma JS. Dynamics of Mycobacterium and bovine tuberculosis in a human-buffalo population. Computational and mathematical methods in medicine. 2014 Jan 1;2014.
60. Hay SI, Rogers DJ, Shanks GD, Myers MF, Snow RW. Malaria early warning in Kenya. Trends in parasitology. 2001 Feb 1;17(2):95-9.
61. Heidenberger K, Flessa S. A system dynamics model for AIDS policy support in Tanzania. European Journal of Operational Research. 1993 Oct 22;70(2):167-76.
62. Hippner P, Sumner T, Houben RM, Cardenas V, Vassall A, Bozzani F, Mudzengi D, Mvusi L, Churchyard G, White RG. Application of provincial data in mathematical modelling to inform sub-national tuberculosis program decision-making in South Africa. PloS one. 2019 Jan 25;14(1):e0209320.
63. Hontelez JA, Lurie MN, Bärnighausen T, Bakker R, Baltussen R, Tanser F, Hallett TB, Newell ML, de Vlas SJ. Elimination of HIV in South Africa through expanded access to antiretroviral therapy: a model comparison study. PLoS Med. 2013 Oct 22;10(10):e1001534.
64. Hontelez JA, Nagelkerke N, Bärnighausen T, Bakker R, Tanser F, Newell ML, Lurie MN, Baltussen R, de Vlas SJ. The potential impact of RV144-like vaccines in rural South Africa: a study using the STDSIM microsimulation model. Vaccine. 2011 Aug 18;29(36):6100-6.
65. Hontelez JA, de Vlas SJ, Baltussen R, Newell ML, Bakker R, Tanser F, Lurie M, Bärnighausen T. The impact of antiretroviral treatment on the age composition of the HIV epidemic in sub-Saharan Africa. AIDS (London, England). 2012 Jul 31;26(0 1).
66. Hontelez JA, De Vlas SJ, Tanser F, Bakker R, Bärnighausen T, Newell ML, Baltussen R, Lurie MN. The impact of the new WHO antiretroviral treatment guidelines on HIV epidemic dynamics and cost in South Africa. PloS one. 2011 Jul 20;6(7):e21919.
67. Hove-Musekwa SD, Nyabadza F, Mambili-Mamboundou H. Modelling hospitalization, home-based care, and individual withdrawal for people living with HIV/AIDS in high prevalence settings. Bulletin of Mathematical Biology. 2011 Dec 1;73(12):2888-915.
68. Hussaini N, Okuneye K, Gumel AB. Mathematical analysis of a model for zoonotic visceral leishmaniasis. Infectious Disease Modelling. 2017 Nov 1;2(4):455-74.
69. Isdory A, Mureithi EW, Sumpter DJ. The impact of human mobility on HIV transmission in Kenya. PloS one. 2015 Nov 24;10(11):e0142805.
70. Johnson LF, Rehle TM, Jooste S, Bekker LG. Rates of HIV testing and diagnosis in South Africa: successes and challenges. Aids. 2015 Jul 17;29(11):1401-9.
71. Johnson LF, May MT, Dorrington RE, Cornell M, Boulle A, Egger M, Davies MA. Estimating the impact of antiretroviral treatment on adult mortality trends in South Africa: A mathematical modelling study. PLoS medicine. 2017 Dec 12;14(12):e1002468.
72. Johnson LF, Davies MA, Moultrie H, Sherman GG, Bland RM, Rehle TM, Dorrington RE, Newell ML. The effect of early initiation of antiretroviral treatment in infants on pediatric AIDS mortality in South Africa: a model-based analysis. The Pediatric infectious disease journal. 2012 May 1;31(5):474-80.
73. Johnson LF, van Rensburg C, Govathson C, Meyer-Rath G. Optimal HIV testing strategies for South Africa: a model-based evaluation of population-level impact and cost-effectiveness. Scientific reports. 2019 Sep 2;9(1):1-2.
74. Johnson LF, Dorrington RE, Moolla H. HIV epidemic drivers in South Africa: A model-based evaluation of factors accounting for inter-provincial differences in HIV prevalence and incidence trends. Southern African journal of HIV medicine. 2017;18(1).
75. Johnson LF, Bekker LG, Dorrington RE. HIV/AIDS vaccination in adolescents would be efficient and practical when vaccine supplies are limited. Vaccine. 2007 Oct 23;25(43):7502-9.
76. Johnson LF, Hallett TB, Rehle TM, Dorrington RE. The effect of changes in condom usage and antiretroviral treatment coverage on human immunodeficiency virus incidence in South Africa: a model-based analysis. Journal of the Royal Society Interface. 2012 Jul 7;9(72):1544-54.
77. Johnson LF, Dorrington RE, Bradshaw D. The role of immunity in the epidemiology of gonorrhoea, chlamydial infection and trichomoniasis: insights from a mathematical model. Epidemiology & Infection. 2011 Dec;139(12):1875-83.
78. Johnson LF, Chiu C, Myer L, Davies MA, Dorrington RE, Bekker LG, Boulle A, Meyer-Rath G. Prospects for HIV control in South Africa: a model-based analysis. Global health action. 2016 Dec 1;9(1):30314.
79. Johnson LF, Stinson K, Newell ML, Bland RM, Moultrie H, Davies MA, Rehle TM, Dorrington RE, Sherman GG. The contribution of maternal HIV seroconversion during late pregnancy and breastfeeding to mother-to-child transmission of HIV. Journal of acquired immune deficiency syndromes (1999). 2012 Apr 1;59(4):417.
80. Johnson LF, Dorrington RE, Moolla H. Progress towards the 2020 targets for HIV diagnosis and antiretroviral treatment in South Africa. Southern African journal of HIV medicine. 2017;18(1).
81. Johnson LF. Access to antiretroviral treatment in South Africa, 2004-2011. Southern African journal of HIV medicine. 2012 Jun 1;13(1).
82. Johnson LF, Dorrington RE, Bradshaw D, Coetzee DJ. The role of sexually transmitted infections in the evolution of the South African HIV epidemic. Tropical Medicine & International Health. 2012 Feb;17(2):161-8.
83. Johnson LF, Dorrington RE, Bradshaw D, Coetzee DJ. The effect of syndromic management interventions on the prevalence of sexually transmitted infections in South Africa. Sexual & Reproductive Healthcare. 2011 Jan 1;2(1):13-20.
84. Johnson LF, Geffen N. A comparison of two mathematical modeling frameworks for evaluating sexually transmitted infection epidemiology. Sexually transmitted diseases. 2016 Mar 1;43(3):139-46.
85. Johnson LF, Dorrington RE, Matthews AP. An investigation into the extent of uncertainty surrounding estimates of the impact of HIV/AIDS in South Africa. South African Journal of Science. 2007 Apr;103(3-4):135-40.
86. Johnson LF, Dorrington RE, Bradshaw D, Pillay-Van Wyk V, Rehle TM. Sexual behaviour patterns in South Africa and their association with the spread of HIV: insights from a mathematical model. Demographic Research. 2009 Jul 1;21:289-340.
87. Johnson LF, Alkema L, Dorrington RE. A Bayesian approach to uncertainty analysis of sexually transmitted infection models. Sexually Transmitted Infections. 2010 Jun 1;86(3):169-74.
88. Johnson LF, Dorrington RE. Modelling the demographic impact of HIV/AIDS in South Africa and the likely impact of interventions. Demographic Research. 2006 Jan 1;14:541-74.
89. Kaabi B, Ahmed SB. Assessing the effect of zooprophylaxis on zoonotic cutaneous leishmaniasis transmission: A system dynamics approach. Biosystems. 2013 Dec 1;114(3):253-60.
90. Kaabi B, Zhioua E. Modeling and comparative study of the spread of zoonotic visceral leishmaniasis from Northern to Central Tunisia. Acta tropica. 2018 Feb 1;178:19-26.
91. Kajunguri D, Hargrove JW, Ouifki R, Mugisha JY, Coleman PG, Welburn SC. Modelling the use of insecticide-treated cattle to control tsetse and Trypanosoma brucei rhodesiense in a multi-host population. Bulletin of Mathematical biology. 2014 Mar 1;76(3):673-96.
92. Kakmeni FM, Guimapi RY, Ndjomatchoua FT, Pedro SA, Mutunga J, Tonnang HE. Spatial panorama of malaria prevalence in Africa under climate change and interventions scenarios. International journal of health geographics. 2018 Dec;17(1):1-3.
93. Kalinda C, Mushayabasa S, Chimbari MJ, Mukaratirwa S. Optimal control applied to a temperature dependent schistosomiasis model. Biosystems. 2019 Jan 1;175:47-56.
94. Kendall EA, Azman AS, Maartens G, Boulle A, Wilkinson RJ, Dowdy DW, Rangaka MX. Projected population-wide impact of antiretroviral therapy-linked isoniazid preventive therapy in a high-burden setting. AIDS (London, England). 2019 Mar 1;33(3):525.
95. Killeen GF, Smith TA, Ferguson HM, Mshinda H, Abdulla S, Lengeler C, Kachur SP. Preventing childhood malaria in Africa by protecting adults from mosquitoes with insecticide-treated nets. PLoS Med. 2007 Jul 3;4(7):e229.
96. Killeen GF, Smith TA. Exploring the contributions of bed nets, cattle, insecticides and excitorepellency to malaria control: a deterministic model of mosquito host-seeking behaviour and mortality. Transactions of the Royal Society of Tropical Medicine and Hygiene. 2007 Sep 1;101(9):867-80.
97. Killeen GF, Ross A, Smith T. Infectiousness of malaria-endemic human populations to vectors. The American journal of tropical medicine and hygiene. 2006 Aug 1;75(2_suppl):38-45.
98. Killeen GF, McKenzie FE, Foy BD, Bøgh C, Beier JC. The availability of potential hosts as a determinant of feeding behaviours and malaria transmission by African mosquito populations. Transactions of the Royal Society of Tropical Medicine and Hygiene. 2001 Sep 1;95(5):469-76.
99. Kimani T, Schelling E, Bett B, Ngigi M, Randolph T, Fuhrimann S. Public health benefits from livestock rift valley fever control: a simulation of two epidemics in Kenya. EcoHealth. 2016 Dec 1;13(4):729-42.
100. Kinyanjui T, Pan-Ngum W, Saralamba S, Taylor S, White L, Nokes DJ. Model evaluation of target product profiles of an infant vaccine against respiratory syncytial virus (RSV) in a developed country setting. Vaccine: X. 2020 Apr 9;4:100055.
101. Kivuti-Bitok LW, McDonnell G, Abdul R, Pokhariyal GP. System dynamics model of cervical cancer vaccination and screening interventions in Kenya. Cost Effectiveness and Resource Allocation. 2014 Dec 1;12(1):26.
102. Kiware SS, Chitnis N, Moore SJ, Devine GJ, Majambere S, Merrill S, Killeen GF. Simplified models of vector control impact upon malaria transmission by zoophagic mosquitoes. PLoS one. 2012 May 31;7(5):e37661.
103. Kokomo E, Danhrée B, Emvudu Y. Mathematical analysis and optimal control of a cholera epidemic in different human communities with individuals’ migration. Nonlinear Analysis: Real World Applications. 2020 Aug 1;54:103100.
104. Kombe IK, Munywoki PK, Baguelin M, Nokes DJ, Medley GF. Model-based estimates of transmission of respiratory syncytial virus within households. Epidemics. 2019 Jun 1;27:1-1.
105. Landouré A, Dembélé R, Goita S, Kané M, Tuinsma M, Sacko M, Toubali E, French MD, Keita AD, Fenwick A, Traoré MS. Significantly reduced intensity of infection but persistent prevalence of schistosomiasis in a highly endemic region in Mali after repeated treatment. PLoS Negl Trop Dis. 2012 Jul 31;6(7):e1774.
106. Lekone PE. Bayesian analysis of severe acute respiratory syndrome: The 2003 Hong Kong epidemic. Biometrical Journal: Journal of Mathematical Methods in Biosciences. 2008 Aug;50(4):597-607.
107. Levy B, Edholm C, Gaoue O, Kaondera-Shava R, Kgosimore M, Lenhart S, Lephodisa B, Lungu E, Marijani T, Nyabadza F. Modeling the role of public health education in Ebola virus disease outbreaks in Sudan. Infectious Disease Modelling. 2017 Aug 1;2(3):323-40.
108. Lilian RR, Johnson LF, Moolla H, Sherman GG. A mathematical model evaluating the timing of early diagnostic testing in HIV-exposed infants in South Africa. JAIDS Journal of Acquired Immune Deficiency Syndromes. 2014 Nov 1;67(3):341-8.
109. Lopman BA, Nyamukapa C, Hallett TB, Mushati P, Spark-du Preez N, Kurwa F, Wambe M, Gregson S. Role of widows in the heterosexual transmission of HIV in Manicaland, Zimbabwe, 1998–2003. Sexually transmitted infections. 2009 Apr 1;85(Suppl 1):i41-8.
110. Lopman B, Gregson S. When did HIV incidence peak in Harare, Zimbabwe? Back-calculation from mortality statistics. PLoS One. 2008 Mar 5;3(3):e1711.
111. Losio AA, Mushayabasa S. Modeling the Effects of Spatial Heterogeneity and Seasonality on Guinea Worm Disease Transmission. Journal of Applied Mathematics. 2018 Jul 5;2018.
112. Lourenço J, Obolski U, Swarthout TD, Gori A, Bar-Zeev N, Everett D, Kamng’Ona AW, Mwalukomo TS, Mataya AA, Mwansambo C, Banda M. Determinants of high residual post-PCV13 pneumococcal vaccine-type carriage in Blantyre, Malawi: a modelling study. BMC medicine. 2019 Dec;17(1):1-1.
113. MacDonald G, Cuellar CB, Foll CV. The dynamics of malaria. Bulletin of the World Health Organization. 1968;38(5):743.
114. Mahiane SG, Nguéma EP, Pretorius C, Auvert B. Mathematical models for coinfection by two sexually transmitted agents: the human immunodeficiency virus and herpes simplex virus type 2 case. Journal of the Royal Statistical Society: Series C (Applied Statistics). 2010 Aug;59(4):547-72.
115. Mahiane SG, Fiamma A, Auvert B. Mixture models for calibrating the BED for HIV incidence testing. Statistics in medicine. 2014 May 10;33(10):1767-83.
116. Maleta K, Bowie C. Selecting HIV infection prevention interventions in the mature HIV epidemic in Malawi using the mode of transmission model. BMC health services research. 2010 Dec 1;10(1):243.
117. Maliyoni M, Chirove F, Gaff HD, Govinder KS. A stochastic tick-borne disease model: Exploring the probability of pathogen persistence. Bulletin of Mathematical Biology. 2017 Sep 1;79(9):1999-2021.
118. Malunguza NJ, Hove-Musekwa SD, Mukandavire Z. Projecting the impact of anal intercourse on HIV transmission among heterosexuals in high HIV prevalence settings. Journal of theoretical biology. 2018 Jan 21;437:163-78.
119. Marx FM, Yaesoubi R, Menzies NA, Salomon JA, Bilinski A, Beyers N, Cohen T. Tuberculosis control interventions targeted to previously treated people in a high-incidence setting: a modelling study. The Lancet Global Health. 2018 Apr 1;6(4):e426-35.
120. Mengistu Kelemu A, Witbooi PJ. Modeling the Effects of Vaccination and Treatment on Tuberculosis Transmission Dynamics. Journal of Applied Mathematics. 2019 Dec 23;2019.
121. Mnyone LL, Lyimo IN, Lwetoijera DW, Mpingwa MW, Nchimbi N, Hancock PA, Russell TL, Kirby MJ, Takken W, Koenraadt CJ. Exploiting the behaviour of wild malaria vectors to achieve high infection with fungal biocontrol agents. Malaria journal. 2012 Dec 1;11(1):87.
122. Moiroux N, Damien GB, Egrot M, Djenontin A, Chandre F, Corbel V, Killeen GF, Pennetier C. Human exposure to early morning Anopheles funestus biting behavior and personal protection provided by long-lasting insecticidal nets. PloS one. 2014 Aug 12;9(8):e104967.
123. Mukhtar AY, Munyakazi JB, Ouifki R, Clark AE. Modelling the effect of bednet coverage on malaria transmission in South Sudan. PloS one. 2018 Jun 7;13(6):e0198280.
124. Mukhtar AY, Munyakazi JB, Ouifki R. Assessing the role of climate factors on malaria transmission dynamics in South Sudan. Mathematical biosciences. 2019 Apr 1;310:13-23.
125. Mukhtar AY, Munyakazi JB, Ouifki R. Assessing the role of human mobility on malaria transmission. Mathematical Biosciences. 2020 Feb 1;320:108304.
126. Muller G, Grébaut P, Gouteux JP. An agent-based model of sleeping sickness: simulation trials of a forest focus in southern Cameroon. Comptes rendus biologies. 2004 Jan 1;327(1):1-1.
127. Musa SS, Zhao S, Gao D, Lin Q, Chowell G, He D. Mechanistic modelling of the large-scale Lassa fever epidemics in Nigeria from 2016 to 2019. Journal of Theoretical Biology. 2020 Feb 22:110209.
128. Mwasa A, Tchuenche JM. Mathematical analysis of a cholera model with public health interventions. Biosystems. 2011 Sep 1;105(3):190-200.
129. Mwesigwa J, Slater H, Bradley J, Saidy B, Ceesay F, Whittaker C, Kandeh B, Nkwakamna D, Drakeley C, Van Geertruyden JP, Bousema T. Field performance of the malaria highly sensitive rapid diagnostic test in a setting of varying malaria transmission. Malaria journal. 2019 Dec 1;18(1):288.
130. Nadhem S, Nejib HD. The Ebola contagion and forecasting virus: evidence from four African countries. Health economics review. 2015 Dec;5(1):1-5.
131. Nagelkerke NJ, Plummer FA, Holton D, Anzala AO, Manji F, Ngugi EN, Moses S. Transition dynamics of HIV disease in a cohort of African prostitutes: a Markov model approach. AIDS (London, England). 1990 Aug;4(8):743-7.
132. Nagelkerke N, Abu-Raddad LJ, Awad SF, Black V, Williams B. A signature for biological heterogeneity in susceptibility to HIV infection?. Infectious Disease Modelling. 2018 Jan 1;3:139-44.
133. Nagelkerke NJ, Moses S, Embree JE, Jenniskens F, Plummer FA. The duration of breastfeeding by HIV-1-infected mothers in developing countries: balancing benefits and risks. JAIDS Journal of Acquired Immune Deficiency Syndromes. 1995 Feb 1;8(2):176-81.
134. Nagelkerke NJ, de Vlas SJ, Jha P, Luo M, Plummer FA, Kaul R. Heterogeneity in host HIV susceptibility as a potential contributor to recent HIV prevalence declines in Africa. AIDS (London, England). 2009 Jan 2;23(1):125.
135. Nannyonga B, Sumpter DJ, Mugisha JY, Luboobi LS. The dynamics, causes and possible prevention of hepatitis E outbreaks. PloS one. 2012 Jul 24;7(7):e41135.
136. Ngarakana-Gwasira ET, Bhunu CP, Masocha M, Mashonjowa E. Assessing the role of climate change in malaria transmission in Africa. Malaria research and treatment. 2016;2016.
137. Nguefack HL, Gwet H, Desmonde S, Oukem-Boyer OO, Nkenfou C, Téjiokem M, Tchendjou P, Domkam I, Leroy V, Alioum A. Estimating mother-to-child HIV transmission rates in Cameroon in 2011: a computer simulation approach. BMC infectious diseases. 2015 Dec 1;16(1):11.
138. Nyabadza F, Bonyah E. On the transmission dynamics of Buruli ulcer in Ghana: Insights through a mathematical model. BMC research notes. 2015 Dec;8(1):1-5.
139. Nyabadza F, Mukandavire Z. Modelling HIV/AIDS in the presence of an HIV testing and screening campaign. Journal of theoretical biology. 2011 Jul 7;280(1):167-79.
140. Nyabadza F, Winkler D. A simulation age-specific tuberculosis model for the Cape Town metropole. South African Journal of Science. 2013 Jan;109(9-10):01-7.
141. Nyabadza F, Mukandavire Z, Hove-Musekwa SD. Modelling the HIV/AIDS epidemic trends in South Africa: Insights from a simple mathematical model. Nonlinear Analysis: Real World Applications. 2011 Aug 1;12(4):2091-104.
142. Ojal J, Flasche S, Hammitt LL, Akech D, Kiti MC, Kamau T, Adetifa I, Nurhonen M, Scott JA, Auranen K. Sustained reduction in vaccine-type invasive pneumococcal disease despite waning effects of a catch-up campaign in Kilifi, Kenya: a mathematical model based on pre-vaccination data. Vaccine. 2017 Aug 16;35(35):4561-8.
143. Ojal J, Griffiths U, Hammitt LL, Adetifa I, Akech D, Tabu C, Scott JA, Flasche S. Sustaining pneumococcal vaccination after transitioning from Gavi support: a modelling and cost-effectiveness study in Kenya. The Lancet Global Health. 2019 May 1;7(5):e644-54.
144. Okango E, Mwambi H, Ngesa O. Spatial modeling of HIV and HSV-2 among women in Kenya with spatially varying coefficients. BMC public health. 2016 Dec 1;16(1):355.
145. Okumu FO, Govella NJ, Moore SJ, Chitnis N, Killeen GF. Potential benefits, limitations and target product-profiles of odor-baited mosquito traps for malaria control in Africa. PLoS One. 2010 Jul 14;5(7):e11573.
146. Omondi EO, Mbogo RW, Luboobi LS. Mathematical modelling of the impact of testing, treatment and control of HIV transmission in Kenya. Cogent Mathematics & Statistics. 2018 Jan 1;5(1):1475590.
147. Omondi EO, Mbogo RW, Luboobi LS. A mathematical modelling study of HIV infection in two heterosexual age groups in Kenya. Infectious Disease Modelling. 2019 Jan 1;4:83-98.
148. Omondi EO, Mbogo RW, Luboobi LS. Modelling the trend of HIV transmission and treatment in Kenya. International Journal of Applied and Computational Mathematics. 2018 Oct 1;4(5):123.
149. Omondi EO, Orwa TO, Nyabadza F. Application of optimal control to the onchocerciasis transmission model with treatment. Mathematical Biosciences. 2018 Mar 1;297:43-57.
150. Omondi EO, Mbogo RW, Luboobi LS. Mathematical analysis of sex-structured population model of HIV infection in Kenya. Letters in Biomathematics. 2018 Dec 14;5(1):174-94.
151. Otiende V, Achia T, Mwambi H. Bayesian modeling of spatiotemporal patterns of TB-HIV co-infection risk in Kenya. BMC infectious diseases. 2019 Dec 1;19(1):902.
152. Pearson CA, Abbas KM, Clifford S, Flasche S, Hladish TJ. Serostatus testing and dengue vaccine cost–benefit thresholds. Journal of the Royal Society Interface. 2019 Aug 30;16(157):20190234.
153. Pretorius C, Stover J, Bollinger L, Bacaër N, Williams B. Evaluating the cost-effectiveness of pre-exposure prophylaxis (PrEP) and its impact on HIV-1 transmission in South Africa. PloS one. 2010 Nov 5;5(11):e13646.
154. Qureshi S, Atangana A. Mathematical analysis of dengue fever outbreak by novel fractional operators with field data. Physica A: Statistical Mechanics and its Applications. 2019 Jul 15;526:121127.
155. Sartorius B, Cohen C, Chirwa T, Ntshoe G, Puren A, Hofman K. Identifying high-risk areas for sporadic measles outbreaks: lessons from South Africa. Bulletin of the World Health Organization. 2013;91:174-83.
156. Shiri T, Auranen K, Nunes MC, Adrian PV, van Niekerk N, de Gouveia L, von Gottberg A, Klugman KP, Madhi SA. Dynamics of pneumococcal transmission in vaccine-naive children and their HIV-infected or HIV-uninfected mothers during the first 2 years of life. American journal of epidemiology. 2013 Dec 1;178(11):1629-37.
157. Silal SP, Little F, Barnes KI, White LJ. Towards malaria elimination in Mpumalanga, South Africa: a population-level mathematical modelling approach. Malaria journal. 2014 Dec 1;13(1):297.
158. Silal SP, Little F, Barnes KI, White LJ. Predicting the impact of border control on malaria transmission: a simulated focal screen and treat campaign. Malaria journal. 2015 Dec 1;14(1):268.
159. Silhol R, Gregson S, Nyamukapa C, Mhangara M, Dzangare J, Gonese E, Eaton JW, Case KK, Mahy M, Stover J, Mugurungi O. Empirical validation of the UNAIDS Spectrum model for subnational HIV estimates: case-study of children and adults in Manicaland, Zimbabwe. Aids. 2017 Apr 1;31(1):S41-50.
160. Ssebuliba DM, Ouifki R, Pretorius C, Burnett SM, Mbonye MK, Naikoba S, Willis K, Weaver MR. Evaluating the impact of two training interventions to improve diagnosis and case-management of malaria and pneumonia in Uganda. Epidemiology & Infection. 2017 Jan;145(1):194-207.
161. Steen R, Hontelez JA, Veraart A, White RG, de Vlas SJ. Looking upstream to prevent HIV transmission: can interventions with sex workers alter the course of HIV epidemics in Africa as they did in Asia?. Aids. 2014 Mar 27;28(6):891-9.
162. Sumaye R, Jansen F, Berkvens D, De Baets B, Geubels E, Thiry E, Krit M. Rift Valley fever: An open-source transmission dynamics simulation model. PloS one. 2019 Jan 9;14(1):e0209929.
163. Torr SJ, Vale GA. Is the even distribution of insecticide-treated cattle essential for tsetse control? Modelling the impact of baits in heterogeneous environments. PLoS Negl Trop Dis. 2011 Oct 18;5(10):e1360.
164. Tulu TW, Tian B, Wu Z. Modeling the effect of quarantine and vaccination on Ebola disease. Advances in Difference Equations. 2017 Dec;2017(1):1-4.
165. Tulu TW, Tian B, Wu Z. Mathematical modeling, analysis and Markov Chain Monte Carlo simulation of Ebola epidemics. Results in physics. 2017 Jan 1;7:962-8.
166. Van den Bergh F, Holloway JP, Pienaar M, Koen R, Elphinstone CD, Woodborne S. A comparison of various modelling approaches applied to Cholera case data. ORiON. 2008;24(1):17-36.
167. van Schalkwyk C, Moodley J, Welte A, Johnson LF. Are associations between HIV and human papillomavirus transmission due to behavioural confounding or biological effects?. Sexually transmitted infections. 2019 Mar 1;95(2):122-8.
168. van Schalkwyk C, Moodley J, Welte A, Johnson LF. Estimated impact of human papillomavirus vaccines on infection burden: The effect of structural assumptions. Vaccine. 2019 Aug 23;37(36):5460-5.
169. Vandormael A, Dobra A, Bärnighausen T, de Oliveira T, Tanser F. Incidence rate estimation, periodic testing and the limitations of the mid-point imputation approach. International Journal of Epidemiology. 2018 Feb 1;47(1):236-45.
170. Vermeulen M, van den Berg K, Sykes W, Reddy R, Ingram C, Poole C, Custer B. Health economic implications of testing blood donors in South Africa for HTLV 1 & 2 infection. Vox sanguinis. 2019 Jul;114(5):467-77.
171. Vickerman P, Ndowa F, O'Farrell N, Steen R, Alary M, Delany-Moretlwe S. Using mathematical modelling to estimate the impact of periodic presumptive treatment on the transmission of sexually transmitted infections and HIV among female sex workers. Sexually transmitted infections. 2010 Jun 1;86(3):163-8.
172. Viljoen T, Spoelstra J, Hemerik L, Molenaar J. Modelling the impact of HIV on the populations of South Africa and Botswana. Acta biotheoretica. 2014 Mar 1;62(1):91-108.
173. Williams BG, Cutts FT, Dye C. Measles vaccination policy. Epidemiology & Infection. 1995 Dec;115(3):603-21.
174. Williams BG, Granich R, De Cock KM, Glaziou P, Sharma A, Dye C. Antiretroviral therapy for tuberculosis control in nine African countries. Proceedings of the National Academy of Sciences. 2010 Nov 9;107(45):19485-9.
175. Wood R, Johnstone-Robertson S, Uys P, Hargrove J, Middelkoop K, Lawn SD, Bekker LG. Tuberculosis transmission to young children in a South African community: modeling household and community infection risks. Clinical infectious diseases. 2010 Aug 15;51(4):401-8.
176. Woolhouse ME, Chandiwana SK. Temporal patterns in the epidemiology of schistosome infections of snails: a model for field data. Parasitology. 1990 Apr;100(2):247-53
177. Woolhouse ME, Watts CH, Chandiwana SK. Heterogeneities in transmission rates and the epidemiology of schistosome infection. Proceedings of the Royal Society of London. Series B: Biological Sciences. 1991 Aug 22;245(1313):109-14.
178. Woolhouse ME, Hasibeder G, Chandiwana SK. On estimating the basic reproduction number for Schistosoma haematobium. Tropical Medicine & International Health. 1996 Aug;1(4):456-63.
179. Yé Y, Sauerborn R, Séraphin S, Hoshen M. Using modelling to assess the risk of malarial infection during the dry season, on a local scale in an endemic area of rural Burkina Faso. Annals of Tropical Medicine & Parasitology. 2007 Jul 1;101(5):375-89.
180. Yé Y, Hoshen M, Kyobutungi C, Louis VR, Sauerborn R. Local scale prediction of Plasmodium falciparum malaria transmission in an endemic region using temperature and rainfall. Global health action. 2009 Nov 11;2(1):1923.
181. Younsi FZ, Bounnekar A, Hamdadou D, Boussaid O. SEIR-SW, simulation model of influenza spread based on the Small World network. Tsinghua Science and Technology. 2015 Oct 13;20(5):460-73.
