# Supplementary material for: African based researchers’ output on models for the transmission dynamics of infectious diseases and public health interventions: A scoping review
Source: PLoS One. 2021 May 6;16(5):e0250086. doi: 10.1371/journal.pone.0250086 (PMC8101744; doi:10.1371/journal.pone.0250086)
Supplement: S2 Table — (DOCX) [file pone.0250086.s003.docx]

|  | **S2 Table. List of African institutional affiliations and count** | | |  |  |
| --- | --- | --- | --- | --- | --- |
|  | **First author’s affiliations** | **Order of authorship** | | | |
|  | **African institutions** | **First** | **Second** | **Third** | **Total** |
| 1 | A.M. Dogliotti College of Medicine, University of Liberia, Monrovia |  |  | 1 | 1 |
| 2 | Addis Ababa Science and Technology University, Addis Ababa | 2 |  |  | 2 |
| 3 | Africa Centre for Health and Population Studies, University of KwaZulu-Natal, Mtubatuba | 5 |  |  | 5 |
| 4 | Africa Health Research Institute, KwaZulu-Natal | 1 |  |  | 1 |
| 5 | African Institute for Mathematical Sciences, Biriwa |  | 1 |  | 1 |
| 6 | African Population and Health Research Centre, Nairobi | 2 |  |  | 2 |
| 7 | ARC – Onderstepoort Veterinary Institute, Pretoria | 1 |  |  | 1 |
| 8 | Biomedical and Environmental Group, Ifakara Health Institute, Ifakara | 3 |  |  | 3 |
| 9 | Center for the Study and Control of Communicable Diseases, Yaoundé | 1 |  |  | 1 |
| 10 | Centre de Recherche en Entomologie de Cotonou, Ministe`re de la Sante´ , Cotonou |  | 1 |  | 1 |
| 11 | Centre for Actuarial Research, University of Cape Town | 4 |  |  | 4 |
| 12 | Centre for Health Policy, Faculty of Health Sciences, School of Public Health, University of Witwatersrand | 1 |  |  | 1 |
| 13 | Centre for Infectious Disease Epidemiology and Research, University of Cape Town, Cape Town | 15 |  |  | 15 |
| 14 | Centre for Sexual Health and HIV AIDS Research, Harare | 1 |  |  | 1 |
| 15 | Clinical Studies Department, School of Veterinary Medicine, University of Zambia, Lusaka | 1 |  |  | 1 |
| 16 | College of Health Sciences, Howard Campus, University of KwaZulu-Natal, Durban | 1 |  |  | 1 |
| 17 | College of Veterinary Medicine and Agriculture, Addis Ababa University, Debre Zeit | 1 |  |  | 1 |
| 18 | Communicable Diseases Research Unit, Blair Research Laboratory, Harare | 1 |  |  | 1 |
| 19 | Community-Based Initiative, Ministry of Health, Monrovia | 1 |  |  | 1 |
| 20 | Complex Systems and Theoretical Biology Group, Laboratory of Research on Advanced Materials and Nonlinear Science, Department of Physics, Faculty of Science, University of Buea, Buea | - | 2 |  | 2 |
| 21 | Department of Agricultural Economics & Agribusiness, Egerton University, Njoro | 1 |  |  | 1 |
| 22 | Department of Applied Mathematics, National University of Science and Technology, Bulawayo | 2 |  |  | 2 |
| 23 | Department of Community Health, University of Nairobi, Nairobi |  | 1 |  | 1 |
| 24 | Department of Computer Science, Faculty of Sciences, University of Oran, Oran | 1 |  |  | 1 |
| 25 | Department of Epidemiology and Biostatistics, Institute of Public Health, Makerere University, Kampala | 1 |  |  | 1 |
| 26 | Department of Mathematical Sciences, Bayero University Kano, Kano | 1 |  |  | 1 |
| 27 | Department of Mathematical Sciences, Pan African University Institute of Basic Sciences Technology and Innovation, Nairobi | 1 |  |  | 1 |
| 28 | Department of Mathematical Sciences, Stellenbosch University, Stellenbosch | 4 |  | 1 | 5 |
| 29 | Department of Mathematics and Applied Mathematics, North-West University, Potchefstroom | 1 |  |  | 1 |
| 30 | Department of Mathematics and Applied Mathematics, University of Pretoria, Pretoria | 2 |  |  | 2 |
| 31 | Department of Mathematics and Applied Mathematics, University of the Western Cape, Bellville | 3 | 2 |  | 5 |
| 32 | Department of Mathematics and Computer Sciences, University of Dschang, Dschang | - | 1 |  | 1 |
| 33 | Department of Mathematics, Federal University Oye Ekiti, Ekiti State | 1 |  |  | 1 |
| 34 | Department of Mathematics, Islamic University in Uganda, Mbale | 1 |  |  | 1 |
| 35 | Department of Mathematics, Kwame Nkrumah University of Science and Technology, Kumasi | 1 |  |  | 1 |
| 36 | Department of Mathematics, Makerere University, Kampala | 2 |  |  | 2 |
| 37 | Department of Mathematics, University of Nigeria, Nsukka |  | 1 |  | 1 |
| 38 | Department of Mathematics, University of Zimbabwe, Harare | 2 |  |  | 2 |
| 39 | Department of Medical Microbiology, University of Nairobi, Nairobi | 2 |  |  | 2 |
| 40 | Department of Production Animal Studies, University of Pretoria | 1 |  |  | 1 |
| 41 | Department of Public Health, Division of Community Health, College of Medicine, University of Malawi, Blantyre | 1 |  |  | 1 |
| 42 | Department of Quantitative Methods, Faculty of Economics and Management of Sfax, Sfax | 1 |  |  | 1 |
| 43 | Department of Statistical Sciences, University of Cape Town, Cape Town | 2 |  |  | 2 |
| 44 | Desmond Tutu HIV Centre, Institute of Infectious Diseases and Molecular Medicine, University of Cape Town, Cape Town | 1 |  |  | 1 |
| 45 | Desmond Tutu TB Centre, Department of Paediatrics and Child Health, Stellenbosch University, Cape Town | 1 |  |  | 1 |
| 46 | Division of Epidemiology and Biostatistics, School of Public Health, Faculty of Health Sciences, University of the Witwatersrand, Johannesburg | 1 |  |  | 1 |
| 47 | DST-NRF Centre of Excellence in Epidemiological Modelling and Analysis, University of Stellenbosch, Stellenbosch | 7 | 3 |  | 10 |
| 48 | DST-NRF: Vaccine Preventable Diseases, University of the Witwatersrand, Johannesburg | - | 1 |  | 1 |
| 49 | DST-NRF Centre of Excellence in Mathematical and Statistical Sciences, University of the Witwatersrand, Gauteng | - | 1 |  | 1 |
| 50 | Ecole Polytechnique, Yaoundé | 1 |  |  | 1 |
| 51 | Epidemiology Research Unit, Johannesburg | 1 |  |  | 1 |
| 52 | Faculty of Mathematical Sciences, University of Khartoum, Khartoum | 1 |  |  | 1 |
| 53 | Health Economics and Epidemiology Research Office, University of the Witwatersrand, Johannesburg | - | 1 |  | 1 |
| 54 | Human Health Division, International Center of Insect Physiology and Ecology, Nairobi | 2 |  |  | 2 |
| 55 | Ifakara Health Institute, Ifakara | 1 |  |  | 1 |
| 56 | Ifakara Health Research and Development Centre, Ifakara | 3 |  |  | 3 |
| 57 | Institut de Recherches en Elevage pour le DeÂveloppement, N'Djameana | 1 |  |  | 1 |
| 58 | Institut National de Recherche en Sante´ Publique, Bamako | 1 |  |  | 1 |
| 59 | Institut Pasteur de Dakar, Dakar | 1 |  |  | 1 |
| 60 | Institut Pasteur de Tunis, Tunis | 1 |  |  | 1 |
| 61 | Institute of Mathematical Sciences, Strathmore University, Nairobi | 5 |  |  | 5 |
| 62 | Institute of Systems Science, Durban University of Technology, Durban | 1 |  |  | 1 |
| 63 | International Centre of Insect Physiology and Ecology, Nairobi | 1 |  |  | 1 |
| 64 | International Livestock Research Institute, Nairobi |  | 1 |  | 1 |
| 65 | International University of East Africa, Kampala | 1 |  |  | 1 |
| 66 | KEMRI-Wellcome Trust Research Programme, Centre for Geographic Medical-Coast, Kilifi | 2 |  |  | 2 |
| 67 | Laboratory of Applied Mathematics, Department of Mathematics and Computer Science, Faculty of Science, University of Douala, Douala | 2 |  |  | 2 |
| 68 | Laboratory of Epidemiology and Veterinary Microbiology, Institut Pasteur de Tunis, Tunis | 1 |  |  | 1 |
| 69 | Laboratory of Medical Epidemiology, Pasteur Institute of Tunis, Tunis | 1 |  |  | 1 |
| 70 | Mathematics Department, University of Botswana, Gaborone | 1 |  |  | 1 |
| 71 | Medical Research Council Unit The Gambia at London School of Hygiene and Tropical Medicine, Banjul | 1 |  |  | 1 |
| 72 | Ministerio das Obras Publicas, Maputo | 1 |  |  | 1 |
| 73 | MIVEGEC (IRD 224-CNRS 5290-UM1-UM2), Institut de Recherche pour le De´veloppement, Cotonou | 1 |  |  | 1 |
| 74 | National Advanced School of Engineering, The University of Yaoundé I, Yaoundé | 1 |  |  | 1 |
| 75 | National Institute of Allergy and Infectious Diseases, PREVAIL-III Study, Monrovia, Liberia | - | 1 |  | 1 |
| 76 | Nelson R Mandela School of Medicine, College of Health Sciences, University of KwaZulu-Natal | - | 1 |  | 1 |
| 77 | Paediatric HIV Diagnostic Syndicate, Wits Health Consortium, Johannesburg | 1 |  |  | 1 |
| 78 | Pan African University Institute for Basic Science, Technology and Innovation, Nairobi | 1 |  |  | 1 |
| 79 | Programme PAC-CI, CHU de Treichville, Abidjan | 1 |  |  | 1 |
| 80 | Research Unit, Foundation for Professional Development, Pretoria | 2 |  |  | 2 |
| 81 | Respiratory and Meningeal Pathogens Research Unit, Chris Hani Baragwanath Hospital, Johannesburg | 1 |  |  | 1 |
| 82 | Sanlam Life Insurance Limited, Bellville, Cape Town | 1 |  |  | 1 |
| 83 | School of Mathematical Sciences, University of KwaZulu-Natal, Durban | 3 | 1 |  | 4 |
| 84 | School of Mathematics, Statistics and Computer Science, University of KwaZulu-Natal, Durban | 2 |  |  | 2 |
| 85 | School of Nursing Sciences, University of Nairobi, Nairobi | 1 |  |  | 1 |
| 86 | The Aurum Institute, Johannesburg | 1 |  |  | 1 |
| 87 | The University Institute of Technology, University of Ngaoundere, Ngaoundere | 1 |  |  | 1 |
| 88 | Tsetse Control Branch, Department of Veterinary Services, Harare | 1 |  |  | 1 |
| 89 | Tugela Ferry Care and Research Collaboration, Tugela Ferry, KwaZulu-Natal | 2 |  |  | 2 |
| 90 | University of Botswana, Gaborone | 1 |  |  | 1 |
| 91 | University of Ngaoundéré, School of Veterinary Medicine and Sciences, Ngaoundéré | 1 |  |  | 1 |

|  | **Last author’s affiliations** | **Order of authorship** | | | |
| --- | --- | --- | --- | --- | --- |
|  | **African institutions** | **First** | **Second** | **Third** | **Total** |
| 1 | Africa Centre for Health and Population Studies, University of KwaZulu-Natal, Durban | 1 |  |  | 1 |
| 2 | Africa Health Research Institute, KwaZulu-Natal | 1 |  |  | 1 |
| 3 | African Institute of Mathematical Sciences, Muizenburg | 1 |  |  | 1 |
| 4 | African Programme for Onchocerciasis Control, Ouagadougou | 1 |  |  | 1 |
| 5 | AIDS and TB Unit, Zimbabwe Ministry of Health and Child Welfare, Harare | 1 |  |  | 1 |
| 6 | Biomedical and Environmental Thematic Group, Ifakara Health Institute, Ifakara | 2 |  |  | 2 |
| 7 | Biomedical Research and Training Institute Harare | 4 |  |  | 4 |
| 8 | Blair Research Laboratory, Harare | 3 |  |  | 3 |
| 9 | Burden of Disease Research Unit, South African Medical Research Council, Tygerberg | 2 |  |  | 2 |
| 10 | Centre de Recherche en Entomologie de Cotonou, Ministe`re de la Sante´, Cotonou | - | 1 |  | 1 |
| 11 | Centre for Actuarial Research, University of Cape Town | 3 |  |  | 3 |
| 12 | Centre for HIV and STI, National Institute for Communicable Diseases, Johannesburg | 1 |  |  | 1 |
| 13 | Centre for Infectious Disease Epidemiology and Research, University of Cape Town, Cape Town | 7 |  |  | 7 |
| 14 | Centre for Social Science Research, University of Cape Town, Cape Town | 1 |  |  | 1 |
| 15 | Centre for Statistics in Ecology, Environment and Conservation, University of Cape Town, Cape Town | - | 1 |  | 1 |
| 16 | Centre for the AIDS Programme of Research in South Africa, Durban | 1 | 1 |  | 2 |
| 17 | College of Biological and Physical Sciences, Institute for Climate Change and Adaptation, University of Nairobi, Nairobi | - |  | 1 | 1 |
| 18 | Communicable Diseases Research Unit, Blair Research Laboratory, Harare | 1 |  |  | 1 |
| 19 | Department of Applied Mathematics, National University of Science and Technology, Bulawayo | 1 |  |  | 1 |
| 20 | Department of Biochemistry, Genetics and Microbiology, University of Pretoria, Pretoria | 1 |  |  | 1 |
| 21 | Department of Community Health, University of Nairobi, Nairobi | - | 1 |  | 1 |
| 22 | Department of Geography, Geoinformation and Meteorology, University of Pretoria, Hatfield | - | 1 |  | 1 |
| 23 | Department of Mathematical Sciences, University of Botswana, Gaborone | 1 |  |  | 1 |
| 24 | Department of Mathematical Sciences, University of Stellenbosch, Stellenbosch | 1 |  |  | 1 |
| 25 | Department of Mathematics and Statistics, Kumasi Polytechnic, Kumasi | 1 |  |  | 1 |
| 26 | Department of Mathematics, Makerere University, Kampala | 2 |  |  | 2 |
| 27 | Department of Mathematics, University of Stellenbosch, Stellenbosch | 1 |  |  | 1 |
| 28 | Department of Mathematics, Vaal University of Technology, Vanderbijlpark | 2 |  |  | 2 |
| 29 | Department of Medical Microbiology, University of Nairobi, Nairobi | 3 |  |  | 3 |
| 30 | Department of Medicine, Faculty of Health Sciences, University of the Witwatersrand, Johannesburg |  | 1 |  | 1 |
| 31 | Department of Medicine, University of Cape Town |  | 1 |  | 1 |
| 32 | Department of Molecular Medicine and Haemotology, University of the Witwatersrand, Johannesburg | 1 |  |  | 1 |
| 33 | Department of Paediatrics and Child Health, Faculty of Health Sciences, University of the Witwatersrand, Johannesburg | - | 1 |  | 1 |
| 34 | Department of Physics, University of Zimbabwe, Harare | 1 |  |  | 1 |
| 35 | Department of Statistical Sciences, University of Cape Town, Cape Town | 1 |  |  | 1 |
| 36 | Desmond Tutu HIV Centre, Institute of Infectious Diseases and Molecular Medicine, University of Cape Town, Cape Town | 5 |  |  | 5 |
| 37 | DST-NRF Centre of Excellence in Epidemiological Modelling and Analysis, University of Stellenbosch, Stellenbosch | 5 | 1 |  | 5 |
| 38 | DST-NRF: Vaccine Preventable Diseases, University of the Witwatersrand, Johannesburg | - | 1 |  | 1 |
| 39 | Faculty of Medicine of Tunis, University of Tunis El Manar, Tunis | - | 1 |  | 1 |
| 40 | Global Alliance for Rabies Control SA NPC, Pretoria |  | 1 |  | 1 |
| 41 | Health Economics and Epidemiology Research Office, University of the Witwatersrand, Johannesburg | 2 |  |  | 2 |
| 42 | Helen Keller International, Regional Office for Africa, Dakar | 1 |  |  | 1 |
| 43 | Human Health Division, International Center of Insect Physiology and Ecology, Nairobi | 2 |  |  | 2 |
| 44 | Ifakara Health Research and Development Centre, Ifakara | 2 |  |  | 2 |
| 45 | Independent Consultant, Enugu |  | 1 |  | 1 |
| 46 | Institut de recherche pour le développement, centre de Yaoundé, Yaoundé | 1 |  |  | 1 |
| 47 | Institut Pasteur de Tunis, Tunis | 1 |  |  | 1 |
| 48 | Institute for Groundwater Studies, University of the Free State, Bloemfontein | 1 |  |  | 1 |
| 49 | Institute of Mathematical Sciences, Strathmore University, Nairobi | 4 |  |  | 4 |
| 50 | International Maize and Wheat Improvement Center, Nairobi | - | 1 |  | 1 |
| 51 | Jomo Kenyatta University of Agriculture and Technology, Department of Statistics and Actuarial Sciences, Nairobi | 1 |  |  | 1 |
| 52 | Kenya Medical Research Institute-Wellcome Trust Research Programme, Centre for Geographic Medicine Research-Coast, Kilifi | 1 |  |  | 1 |
| 53 | Laboratoire d’Ecologie Animale Terrestre, Faculté des Sciences Semlalia, Marrakech | 1 |  |  | 1 |
| 54 | Laboratory of Medical Epidemiology, Pasteur Institute of Tunis, Tunis | 1 |  |  | 1 |
| 55 | Laboratory of protein engineering and bioactive molecular, Institut National de Sciences Appliquées et de Technologie, Tunis | 1 |  |  | 1 |
| 56 | Malaria Research and Training Centre, Department of Epidemiology of Parasitic Diseases, Faculty of Medicine, Pharmacy and Odonto-Stomatology, University of Bamako, Mali, Bamako, | 1 |  |  | 1 |
| 57 | Malawi-Liverpool Wellcome Trust Clinical Research Programme, Blantyre | 1 |  |  | 1 |
| 58 | Masoka Management Training Institute and Conference Centre, Moshi | 1 |  |  | 1 |
| 59 | Mathematics and Informatics Department, Taita Taveta University College, Voi |  | 1 |  | 1 |
| 60 | Mathematics Department, University of Dar es Salaam, Dar es Salaam | 1 |  |  | 1 |
| 61 | Medical Research Council Laboratories (UK), Fajara | 1 |  |  | 1 |
| 62 | Medical Research Council Unit The Gambia at London School of Hygiene and Tropical Medicine, Banjul | 1 |  |  | 1 |
| 63 | Metabiota Inc., Kenema Government Hospital, Kenema | 1 |  |  | 1 |
| 64 | MIVEGEC (IRD 224-CNRS 5290-UM1-UM2), Institut de Recherche pour le De´veloppement, Cotonou | 1 |  |  | 1 |
| 65 | National AIDS/STD Control Programme | 1 |  |  | 1 |
| 66 | National Health Laboratory Service | - | 1 |  | 1 |
| 67 | National Institute for Communicable Diseases, National Health Laboratory Service, Sandringham | - |  | 1 | 1 |
| 68 | Noguchi Memorial Institute for Medical Research, University of Ghana, Legon, Accra | 1 |  |  | 1 |
| 69 | Office of Malaria Research, South African Medical Research Council, Durban | 1 |  |  | 1 |
| 70 | Reproductive Health and HIV Research Unit, Johannesburg | 1 |  |  | 1 |
| 71 | Respiratory and Meningeal Pathogens Research Unit, Chris Hani Baragwanath Hospital, Johannesburg | 1 |  |  | 1 |
| 72 | School of Life Sciences, College of Agriculture, Engineering and Science, Westville Campus, University of KwaZulu-Natal, Durban | 1 |  |  | 1 |
| 73 | School of Mathematics, Statistics and Computer Science, University of KwaZulu–Natal | 3 |  |  | 3 |
| 74 | School of Mathematics, University of Nairobi, Nairobi | 1 |  |  | 1 |
| 75 | School of Nursing and Public Health, University of KwaZulu-Natal | - |  | 1 | 1 |
| 76 | School of Physics, University of KwaZulu-Natal, Durban | 1 |  |  | 1 |
| 77 | Social Aspects of HIV/AIDS and Health, Human Sciences Research Council | 1 |  |  | 1 |
| 78 | South African Weather Service, Pretoria | 1 |  |  | 1 |
| 79 | The Faculty of Sciences of University of Ngaoundere, Ngaoundere | 1 |  |  | 1 |
| 80 | Tugela Ferry Care and Research Collaboration, KwaZulu-Natal | 2 |  |  | 2 |
| 81 | University of Juba, Department of Mathematics, Juba, Central Equatoria | 1 |  |  | 1 |
| 82 | University of Ngaoundéré, School of Veterinary Medicine and Sciences, Ngaoundéré | 1 |  |  | 1 |
| 83 | Wellcome Centre for Infectious Diseases Research in Africa, Institute of Infectious Disease and Molecular Medicine, University of Cape Town | 1 |  |  | 1 |
| 84 | Wits Reproductive Health and HIV Institute, University of Witwatersrand, Johannesburg | 1 |  |  | 1 |
| 85 | World Health Organization Field Research Project, Kankiya | 1 |  |  | 1 |
